# A comparison of verbal autopsy assignment methods to obtain adult cause-specific mortality in two longitudinal studies in Rakai and Kalungu districts of South Central, Uganda

**DOI:** 10.1371/journal.pgph.0006223

**Published:** 2026-04-06

**Authors:** Dorean Nabukalu, Tom Lutalo, Joseph Mugisha, Ronald Makanga, Ivan Kasamba, Clara Calvert, Palwasha Khan, Milly Marston, Jim Todd

**Affiliations:** 1 Department of Population Health, London School of Hygiene and Tropical Medicine, London, United Kingdom; 2 Department of Data Management and Statistics, Rakai Health Sciences Program, Rakai, Uganda; 3 Department of Epidemiology, Data and Statistics, Uganda Virus Research Institute, Entebbe, Uganda; 4 Department of Epidemiology, Statistics and Data Science, The Medical Research Council/Uganda Virus Research Institute and the London School of Hygiene & Tropical Medicine, Entebbe, Uganda; 5 Usher Institute, University of Edinburgh, Edinburgh, United Kingdom; 6 Department of Clinical Research, London School of Hygiene and Tropical Medicine, London, United Kingdom; 7 Department of Epidemiology, Biostatistics and Behavioural Sciences, Catholic University for Health and Allied Sciences (CUHAS), Mwanza, Tanzania; PLOS: Public Library of Science, UNITED STATES OF AMERICA

## Abstract

Verbal Autopsy (VA) data determines cause of death (CoD) in settings where certification is lacking, using structured interviews on symptoms and circumstances before death. Various methods are used to interpret this information and assign a probable cause of death. Debate exists over replacing physician reviews with automated methods. We compared physician reviews with two computer algorithms in assigning adult CoD. We used adult (≥15 years) VA data collected at two Health and Demographic Surveillance System (HDSS) sites in Uganda (Rakai and Kyamulibwa) collected between 2013 and 2021. CoD were categorized into six broad groups, and observed cause-specific mortality fractions (CSMF) were calculated. We evaluated the performance of physician reviews and two algorithms (InterVA-5 and InSilicoVA) based on four individual and population-level metrics (CSMF accuracy, percentage agreement, sensitivity, and Spearman’s correlation coefficient). Data from Rakai compared physician reviews and algorithms, while Kyamulibwa data compared the two algorithms. A total of 1564 VA records from Rakai and Kyamulibwa were analysed. In Rakai, InSilicoVA showed higher CSMF for other communicable causes, excluding HIV/TB (males 24.1%, 95% CI:20.1-28.7; females 25.2%, 95% CI: 20.8-30.3) than Physician reviews (males 14.8%, 95% CI: 11.6-18.8; females 17.5%, 95% CI: 13.8-22.1) and InterVA-5 (males 11.1%, 95% CI: 8.3-14.8; females11.7%, 8.6-15.7). Non-communicable diseases CSMF was lowest with InSilicoVA (males 30.5%, 95% CI:26.1-35.4, and females 38.46%, 33.31-43.89) compared to the InterVA-5. The CSMF accuracy and percentage agreement demonstrated comparable performance between physician reviews and computer algorithms, with substantial agreement in identifying causes of death. InSilicoVA was more sensitive for infectious, pregnancy, and external causes, while InterVA-5 better identified non-communicable and HIV/TB-related deaths. Computer algorithms can complement physician review in resource-limited settings, but current VA tools rely on structured symptom data and exclude rich narrative information. Incorporating qualitative information in future algorithms may improve symptom–cause relationships, accuracy, and cause-of-death assignment.

## Introduction

In low- and middle-income countries (LMIC), there are about 55 million deaths annually, of which 45 million (82%) occur at home [1,2]. Deaths that occur in facilities sometimes have clinical, laboratory, and diagnostic records that can be used to assign a cause of death (CoD). However, deaths that occur at home have no medical records, and therefore, it is difficult to assign a CoD [3]. Consequently, there are few direct estimates of cause-specific mortality in LMIC, making it difficult to track progress on international targets to reduce deaths from particular causes [1]. Verbal Autopsy (VA) can be used to assign CoD in countries without robust civil registration vital systems (CRVS), specifically, in settings where medical certification of deaths is uncommon [3,4].

VAs are structured interviews with relatives or carers of deceased individuals, during which data on the signs and symptoms that occurred before death are collected. The tool used to collect these data contains a section where the caregiver responds to semi-structured questions asked by the interviewer (usually a clinically trained person). The tool also contains a narrative section where the caregiver, in their own words, gives an account of the illness and what they think could have caused the death of the deceased. The WHO recommends that these data be collected between four weeks and 12 months after the occurrence of death [5]. Currently, two methods are used to interpret VA data and assign a cause of death: (1) physician reviews (since the early 1990s) and (2) computerized algorithms (since the early 2000s) [6]. Physician-based review involves two independent physicians reviewing the data collected in the VA interview and assigning the most likely cause of death. A third reviewer is consulted to resolve any disagreement between the two independent reviewers. The physician-based review method is preferred by some researchers as physicians can apply their local knowledge of diseases and can take advantage of the narrative component of the VA. However, there has been a shift toward algorithmic approaches to assign CoD, which offer efficiency, scalability, consistency, and cost-effectiveness, particularly in resource-constrained environments, facilitating timely, crucial mortality data for policymaking and healthcare planning [7].

Several computer algorithms have been developed, and currently, three commonly used computerized algorithms for assigning CoD from VA data are available in the public domain. These are InterVA-5, InSilicoVA, and Tariff [8]. Several studies have evaluated and compared the algorithms with physician reviews globally [3,7–11] and in Uganda [12]. The key observation in these studies is that while the computer algorithms remain desirable, no method was able to replicate the physician assignment of COD more than half the time, hence, physician reviews are still preferred (if available) in determining CoD, while the algorithms are being improved. While it remains a contentious issue whether computer algorithms can replace physician reviews, many studies using VA have moved to relying on algorithms, limiting opportunities to compare these different methods.

This study draws on mortality data from two Ugandan population cohorts—the Rakai Community Cohort Study (RCCS) and the Kyamulibwa General Population Cohort—to evaluate the performance of two widely used verbal autopsy (VA) algorithms for assigning CoD, InterVA-5 and InSilicoVA-v1.4. For the first objective, we compared algorithm outputs with physician-reviewed verbal autopsies using only the RCCS dataset, as physician review data were available exclusively for this population.. This addressed the need to assess agreement with clinical judgment, which remains a conventional reference despite known limitations such as inter-reviewer variability [13]. The second objective directly compared InterVA-5 and InSilicoVA-v1.4 across both cohorts to examine consistency and generalizability in distinct epidemiological contexts. This dual approach responds to calls for robust algorithm validation across diverse settings, as highlighted in multisite studies by Flaxman et al. [14] and Nichols et al. [8], and contributes to strengthening mortality surveillance in regions where VA remains the primary source of cause-of-death data.

## Materials and methods

### Ethics statement

Before enrolment into the activities of the HDSS, following Uganda National Council of Science and Technology (UNCST) guidelines, all adult participants (aged 18 years and above) at both sites provided written informed consent, while adolescents (aged 15–17 years) provided assent and their parents or guardians provided written consent on their behalf. Each of the HDSS received ethical approval from the different local Institutional Review Boards (IRBs) to collect and regularly update population data. For this project, ethical approval was obtained from the LSHTM ethics board, and data sharing agreements were signed with each of the sites to access the data.

### Study design, setting, and data collection

This study used VA data collected at two sites that run Health and Demographic Surveillance Systems (HDSS) in Uganda: the Rakai Community Cohort Study (known as Rakai) in South Central Uganda, and Kyamulibwa General Population Cohort (known as Kyamulibwa) in Southwest Uganda. Data from Rakai was accessed on 1st March 2024, while data from Kyamulibwa was accessed on 27th March 2024. Both sites use unique study IDs to identify individual participants. The data managers from both sites created and assigned unique ID numbers to individual participants for this study. HDSS sites collect routine data on residency status, births, education, migration, and deaths. They also collect VA data on all deaths in the population, administered by clinically trained research assistants on average within four to six months after death. The HDSS sites are independently managed with site-specific data collection protocols, data collection tools, and data management systems and processes [15]. For this study, all data on adult deaths (15 years and above) were used. We included all deaths that occurred between January 2013 and December 2021 among adult individuals who were registered residents of the demographic surveillance catchment areas and for whom a verbal autopsy (VA) interview was successfully conducted. During this period, both sites consistently employed the standardised WHO 2012 VA instrument, ensuring methodological uniformity across time and location. Deaths were excluded if no VA interview could be obtained or if the respondent refused participation. These inclusion and exclusion criteria were applied uniformly across both sites to maximize data quality and comparability of cause-specific mortality estimates [5]. More details of these HDSS are documented elsewhere [16,17].

### Cause of death assignment

Three methods were used to assign CoD: physician review, InterVA-5 computer algorithm, and InSilicoVA-v1.4 computer algorithm [18,19]. Physician review CoD was only available in Rakai, where two trained physicians independently reviewed the VA data and separately assigned an underlying CoD using ICD-10 codes for disease classification based on the symptoms collected in the VA tool and the narrative describing the circumstances leading up to death from the respondent. Where there was disagreement between the two reviewers, a third physician was consulted, and the three reviewed the VA together and came to a consensus. Failure to assign a cause of death from this meeting resulted in an indeterminate cause of death being assigned [20].

InterVA-5 uses the coded responses from the VA symptoms reported in the WHO standardized VA questionnaire as the inputs. The model uses a list of sixty causes of death created by the model experts from the detailed ICD-10 cause list and assigns a probability to each of the sixty causes of death for each death with VA symptoms. It uses a deterministic algorithm based on a modified Naïve Bayes classifier [18,21], that uses a pre-defined symptom cause information (SCI) matrix that describes the conditional probability of each symptom for each CoD.

InSilicoVA-v1.4 [19] uses the same input (coded symptoms from the WHO standardized VA questionnaire) and outputs (probability of the same sixty-cause list) as InterVA-5. It uses the same SCI matrix as InterVA-5 but uses a full Bayesian framework to assign the probabilities to each CoD. It provides a probabilistic assignment, meaning the predicted cause of death comes with a level of confidence, expressed as a probability or uncertainty.

All deaths were included in the comparison between InterVA-5 and InSilicoVA. However, deaths classified as having an undetermined cause by physician review were excluded from the comparison between physician reviews and the algorithmic outputs, as these lacked a definitive reference diagnosis needed to evaluate agreement with algorithmic outputs. A separate analysis was conducted on these physician-classified undetermined deaths to illustrate how the computer algorithms distributed causes of death in such cases.

### Data preparation

For the physician reviews, every death was assigned an underlying CoD based on the detailed ICD-10 list. The standardized openVA toolkit [22] in R were used to assign CoD for both of the algorithms, InterVA-5 [23] and InSilicoVA-v1.4 [24]. The cause with the highest probability was assigned as the potential CoD, with a cutoff probability of 0.4. An undetermined CoD was assigned if the highest probability was less than 0.4. This threshold was chosen to represent a moderate level of confidence – high enough to suggest a likely cause of death, but not so strict that other plausible causes are ignored, as suggested by Byass et al. [25].

The Physician reviews and the computer algorithms had different CoD lists. These were reviewed, harmonized, and mapped onto a list of thirty-five causes of death (Harmonised CoD). For this study, when a physician-assigned cause of death did not have a direct match in the algorithm cause list, it was carefully mapped to the most appropriate corresponding category supported by the algorithms, based on the causes available in the data. This mapping process was conducted with attention to preserving clinical meaning and was independently reviewed and validated by a physician to ensure accuracy and consistency (S1 Table). This list was further reclassified into the fourteen specific CoD categories (WHO CoD) defined by WHO in 2012 [5]. HIV and TB were split out from the infectious disease category because these HDSS are located in HIV endemic areas, which have had several interventions to control the HIV epidemic, thus fifteen specific CoD. A table showing how the ICD-10 causes are mapped to the WHO cause list is included as S3 Table. HIV and TB were combined into one category (HIV/TB-related CoD) because HIV and TB are often co-infections. The fifteen specific causes were then grouped into six broad CoD categories: other communicable causes, HIV/TB-related causes, non-communicable causes, maternal causes, external causes, and undetermined causes.

### Statistical analysis

Cause-specific mortality fractions (CSMF) were calculated by dividing the observed number in each cause of death by the total number of deaths with an assigned CoD. CSMF and classical 95% confidence intervals (95%CI) were obtained stratified by age groups (15-29, 30-49, 50+), sex and HDSS site. CSMF was calculated for each method (physician review, InterVA-5, and InSilicoVA-v1.4) and each of the three CoD lists (Harmonised CoD, WHO CoD, and Broad CoD).

### Analysis 1: Comparison of algorithms with physician review in Rakai

For Rakai, where physician review was available, CoD from InterVA-5 and InSilicoVA-v1.4 were compared to Physician review, at the individual and population level. In this comparison, the physician review method was taken as the gold standard in the absence of hospital-based medical certification of death. Physician reviews were further considered because of their expert judgment, as they bring in clinical knowledge and expertise to the process, making them well-equipped to interpret symptoms and medical histories described in the VA interview. Their professional experience in diagnosing causes of death makes their judgments highly valued [19,26]. The most probable cause of death assigned by InterVA-5 and InSilicoVA-v1.4 was used. At the individual level, as metrics, the sensitivity of the algorithms compared to the physician reviews was calculated based on the fifteen-cause list, and the percent agreement between the algorithms and Physician reviews was calculated (considering the thirty-five-cause list and the fifteen-cause list). To assess agreement at the population level, two metrics were calculated: cause-specific mortality fraction accuracy (CSMF accuracy), and Spearman’s rank correlation coefficient. For these metrics, 95% confidence intervals were obtained by non-parametric bootstrap method. These metrics are explained below.

**CSMF accuracy**: While the percent agreement measures the proportion of individual cases where the predicted cause exactly matches the true cause, the CSMF accuracy quantifies how closely the CSMF values of the computer algorithms approximate the CSMF values of the Physician reviews. It measures how well the distribution of causes of death by the computer algorithms (CSMF pred) matches the distribution of causes of death by the physician reviews (CSMF true). The CSMF accuracy captures how close the distribution of predicted causes of death is to the true distribution, even if many individual predictions are wrong. In contrast, percentage agreement only tells you how often the predictions are right on an individual level, ignoring whether the population pattern is accurate.

The CSMF accuracy ranges from 0 to 1, with 0 being the worst case and 1 being the perfect alignment between the CSMFs of the computer algorithms and Physician reviews.


$$CSMF\;accuracy=\text{1}-\frac{\sum_{j=\text{1}}^{k}\left|\overset{true}{\underset{j}{CSMF}}-\;\overset{pred}{\underset{j}{CSMF}}\right|}{\text{2}(\text{1}-Minimum(\overset{true}{\underset{j}{CSMF}}))}$$

**Spearman’s rank correlation coefficient**: This measures the similarities between the rankings of the causes produced by the computer algorithms (predicted) and the rankings produced by the Physician reviews (true ranking). It is computed as:


Rho=corr(Rank(CSMF^predicted), Rank(CSMF^true))

where Rank (CSMF) is the rank of CSMFs, i.e., the cause with the largest CSMF receives a rank of 1 and the cause with the smallest CSMF receives a rank of 6.**Sensitivity**: This is also known as the true positive rate. It was computed and assessed for each category of CoD at an individual level. It was computed as the percentage of deaths in a specific CoD assigned by physician reviews and correctly assigned by the algorithms. Based on the WHO CoD. The following formula was used:


$${Sensitivity}_{j}\;=\;({TP}_{j}/\;({TP}_{j}\;+\;{FN}_{j}))\mathrm{\backslash ]}$$

Where:

- *TP*_*j*_ is true positives (where computer algorithms CoD is the same as the specific CoD assigned by physician reviews).
- *FN*_*j*_ is false negatives (where computer algorithms assign a different CoD to the specific CoD assigned by the physician reviews).

### Analysis 2: Comparison of computer algorithms with one another in Rakai and Kyamulibwa

This comparison was done for data from Rakai and Kyamulibwa. We computed the percent agreement between the algorithms at the individual and broad levels. The percentage of deaths where the two computer algorithms assigned the same CoD was computed and assessed for all CoD and compared between age and sex subgroups. All analyses were conducted using Stata V.18 [27].

## Results

**Fig 1:**
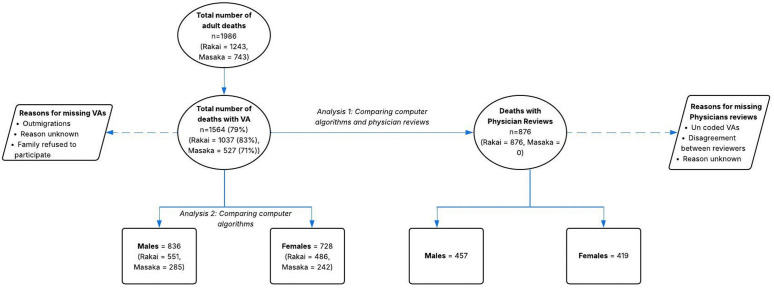
Description of deaths in Rakai and Kyamulibwa.

Between 2013 and 2021 inclusive, a cumulative number of 1986 adult deaths (15 years and above) were reported. Of these, 1,564 (66.1%) had a VA, with 1,037 in Rakai and 527 in Kyamulibwa. Table 1 presents the distribution of deaths by year of VA interviews conducted in Rakai and Masaka. The data indicate that the collection of VA data remained consistent over the years, with no observable disruption attributable to the COVID-19 pandemic. This suggests that the pandemic did not significantly affect the VA interview process in these regions. There were more male deaths (53.5%) (Rakai – 551 (65.9%) and Kyamulibwa – 285 (34.1%)) than female deaths (46.5%) (Rakai – 486 (66.8%) and Kyamulibwa – 242 (33.2%)) as shown in Fig 1. Table 2 shows the breakdown of deaths by sex and age group, with most deaths occurring in those aged 50 + years.

**Table 1:** Deaths by Year of Verbal Autopsy Interview in Rakai and Kyamulibwa.

	Rakai			Kyamulibwa		
VA Interview year	Deaths (n = 1243)	VAs (n = 1037) (%)	PCVA (n = 876) (%)	Deaths (n = 743)	VAs (n = 527) (%)	PCVA
2013	173	142/173 (82)	124/142 (87)	0	0	0
2014	134	108/134 (80)	98/108 (91)	70	55/70 (79)	0
2015	135	111/135 (82)	96/111 (86)	62	44/62 (71)	0
2016	142	117/142 (83)	101/117 (86)	95	76/95 (80)	0
2017	144	115/144 (80)	108/115 (94)	76	54/76 (71)	0
2018	140	110/140 (79)	104/110 (95)	0	0	0
2019	137	113/137 (82)	98/113 (87)	45	25/45 (56)	0
2020	140	124/140 (89)	92/124 (74)	154	116/154 (75)	0
2021	99	97/99 (98)	55/97 (57)	241	157/241 (65)	0

**Table 2:** Sex age distributions of deaths with Verbal Autopsy in Rakai and Kyamulibwa, 2013 to 2021.

	Men			Women		
	^1^Total deaths: n (col%)	^2^Deaths with a VA: n (col%)	^3^Deaths with a VA and a physician review CoD: n (col %)	^4^Total deaths: n (col%)	^5^Deaths with a VA: n (col%)	^6^Deaths with a VA and a physician review CoD: n (col%)
**Rakai**						
15-29 years	83 (12.4)	67 (12.2)	54 (11.8)	77 (13.7)	59 (12.1)	52 (12.4)
30-49 years	218 (32.7)	176 (31.9)	145 (31.7)	127 (22.5)	109 (22.4)	93 (22.2)
50 + years	366 (54.9)	308 (55.9)	258 (56.5)	360 (63.8)	318 (65.4)	274 (65.4)
**Kyamulibwa**						
15-29 years	41 (10.5)	27 (9.5)	0 (0%)	32 (9.1)	19 (7.9)	0 (0%)
30-49 years	58 (14.8)	45 (15.8)	0 (0%)	63 (17.9)	37 (15.3)	0 (0%)
50 + years	292 (74.7)	213 (74.7)	0 (0%)	257 (73.0)	186 (76.9)	0 (0%)

^1^: Rakai (N=667), Masaka (N=391).

^2^: Rakai (N=551), Masaka (N=285).

^3^: Rakai (N = 457).

^4^: Rakai (N = 564), Masaka (N = 352).

^5^: Rakai (N = 486), Masaka (N = 242).

^6^: Rakai (N = 419).

### Analysis 1: Comparison of algorithms with physician review in Rakai

**Fig 2:**
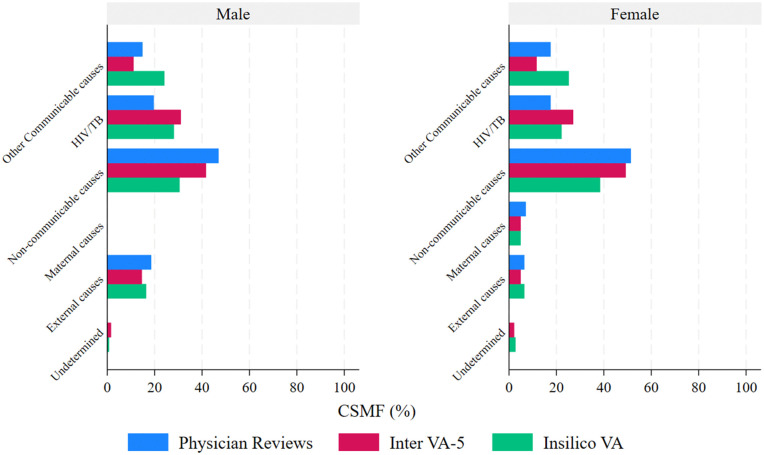
Overall cause-specific mortality fraction at the broad level comparing computer algorithms and Physician reviews stratified by sex in Rakai.

Table 3 and Fig 2 show the leading cause of death as assigned by each method at the individual and population levels for the 876 deaths in Rakai, where a physician review CoD was available. Overall, communicable diseases (other communicable and HIV/TB combined) were consistently the leading causes of death for all three methods. The CSMF of non-communicable diseases was lowest from the InSilicoVA-v1.4 method (males - 30.5%, 95% CI:26.1-35.4, and females – 38.46%, 33.31-43.89) compared to the InterVA-5 (males - 41.64%, 95% CI:36.8-46.7; females - 49.2%, 95% CI:43.8-54.7) and Physician reviews (46.9%, 95% CI:41.9-52.0; females - 51.4%, 95% CI:45.9-56.8). There were small variations in CSMF assigned to other causes of death by the different methods. InSilicoVA-v1.4 assigned substantially more deaths to other communicable causes (excluding HIV/TB) (males - 24.1%, 95% CI:20.1-28.7; females - 25.2%, 95% CI: 20.8-30.3) compared to Physician reviews (males - 14.8%, 95% CI: 11.6-18.8; females -17.5%, 95% CI: 13.8-22.1) and InterVA-5 (males - 11.1%, 95% CI: 8.3-14.8; females -11.7%, 8.6-15.7) for both males and females. The InterVA-5 method assigned more deaths to HIV/TB-related causes (males - 31.0%, 95% CI:26.6-35.9, and females - 27.1%, 95% CI: 22.5-32.2) compared to Physician reviews (males - 19.6%, 95% CI:15.9-23.9 and females - 17.5%, 95% CI:13.8-22.1) and InSilicoVA-v1.4 (males - 28.1%, 95% CI:23.8-32.9 and females - 22.2%, 95% CI:17.9-27.0) (Fig 2 and Table 3). S2 Table shows a classification of the deaths assigned as undetermined by the physician reviews compared to the algorithms. The largest proportion of these deaths are assigned as infectious and parasitic causes by both InterVA-5 (33%) and InSilicoVA-v1.4 (43%); of note, InterVA-5 assigned a higher proportion of these deaths (17%) as neoplasms compared to InSilicoVA-v1.4 (8%).

**Table 3:** Cause-Specific Mortality Fractions (%) in Rakai, stratified by sex and the different COD assignment methods, 2013 to 2021.

Broad cause of death	Specific cod	Males			Females		
		Physician reviews CSMF(95% CI)	InterVA5 CSMF(95% CI)	InSilico VA CSMF(95% CI)	Physician reviews CSMF(95% CI)	InterVA5 CSMF(95% CI)	Insilico VA CSMF(95% CI)
**Communicable diseases**		**14.85(11.60-18.83)**	**11.14(8.33-14.75)**	**24.14(20.07-28.73)**	**17.54(13.77-22.08)**	**11.69(8.62-15.68)**	**25.23(20.79-30.26)**
	Acute respiratory infection including Pneumonia	5.04(3.23-7.78)	4.51(2.82-7.14)	4.77(3.02-7.46)	7.69(5.24-11.15)	6.46(4.24-9.72)	7.69(5.24-11.15)
	Sepsis (non-obstetric)	1.33(0.55-3.15)	0.53(0.13-2.10)	0(-)	1.23(0.46-3.24)	0.62(0.15-2.44)	0(-)
	Diarrhoeal diseases	0(-)	0(-)	0(-)	1.23(0.46-3.24)	0.24(0.03-1.68)	0.24(0.03-1.68)
	Malaria	5.04(3.23-7.78)	0.27(0.04-1.87)	0.79(0.26-2.45)	5.54(3.51-8.63)	0(-)	0.62(0.15-2.44)
	Meningitis and encephalitis	0.79(0.26-2.45)	0.53(0.13-2.10)	1.86(0.89-3.85)	0(-)	0.62(0.15-2.44)	0.62(0.15-2.44)
	Other and unspecified infectious diseases	2.65(1.43-4.87)	5.31(3.44-8.09)	16.71(13.27-20.84)	1.85(0.83-4.06)	4.00(2.33-6.78)	16.31(12.67-20.75)
**HIV/AIDS/TB**		**19.63(15.91-23.96)**	**31.03(26.55-35.90)**	**28.12(23.79-32.88)**	**17.54(13.77-22.08)**	**27.08(22.51-32.19)**	**22.15(17.95-27.01)**
**Non communicable diseases**		**46.95(41.94-52.02)**	**41.64(36.75-46.71)**	**30.50(26.05-35.36)**	**51.38(45.93-56.80)**	**49.23(43.81-54.67)**	**38.46(33.31-43.89)**
	Oral neoplasms	0(-)	0.53(0.13-2.10)	0(-)	0.62(0.15-2.44)	0(-)	0(-)
	Digestive neoplasms	4.24(2.61-6.82)	11.41(8.56-15.04)	7.43(5.17-10.56)	3.38(1.88-6.02)	5.54(3.51-8.63)	4.62(2.79-7.53)
	Respiratory neoplasms	0.53(0.13-2.10)	3.71(2.21-6.18)	1.59(0.71-3.51)	0.31(0.04-2.17)	0.62(0.15-2.44)	0(-)
	Breast neoplasms	0(-)	0(-)	0(-)	0.92(0.29-2.83)	0.62(0.15-2.44)	0.62(0.15-2.44)
	Reproductive neoplasms	2.65(1.43-4.87)	0.27(0.04-1.87)	0(-)	2.15(1.03-4.46)	6.15(3.99-9.36)	3.38(1.88-6.02)
	Other and unspecified neoplasms	5.04(3.23-7.78)	3.71(2.21-6.18)	2.39(1.24-4.53)	2.77(1.44-5.25)	1.54(0.64-3.65)	1.23(0.46-3.24)
	Severe anaemia	0.53(0.13-2.10)	0(-)	0(-)	0(-)	0(-)	0(-)
	Severe malnutrition	0(-)	0(-)	0(-)	0.31(0.04-2.17)	0(-)	0.31(0.04-2.17)
	Diabetes mellitus	4.24(2.61-6.82)	2.12(1.06-4.19)	0.26(0.04-1.87)	3.08(1.66-5.63)	2.46(1.23-4.86)	0.92(0.29-2.83)
	Liver disease	3.18(1.81-5.53)	1.86(0.89-3.85)	1.59(0.71-3.51)	2.77(1.44-5.25)	0.92(0.29-2.83)	1.23(0.46-3.24)
	Acute abdomen	1.86(0.89-3.85)	0.53(0.13-2.10)	3.18(1.81-5.53)	1.23(0.46-3.24)	0.62(0.15-2.44)	1.85(0.83-4.06)
	Renal failure	1.06(0.39-2.80)	2.12(1.06-4.19)	3.44(2.01-5.86)	0.92(0.29-2.83)	2.46(1.23-4.86)	2.77(1.44-5.25)
	Cardiac disease	9.81(7.19-13.27)	13.79(10.66-17.67)	9.55(6.96-12.97)	24.31(19.94-29.29)	27.08(22.51-32.19)	21.23(17.11-26.03)
	Other and unspecified NCD	9.81(7.19-13.27)	0.27(0.04-1.87)	0(-)	4.31(2.56-7.15)	0.24(0.03-1.68)	0.31(0.04-2.17)
	Epilepsy	3.98(2.41-6.50)	1.32(0.55-3.15)	1.06(0.39-2.80)	4.31(2.56-7.15)	1.23(0.46-3.24)	0(-)
**Maternal Causes of death**					**7.08(4.74-10.44)**	**4.92(3.03-7.89)**	**4.92(3.03-7.89)**
	Abortion-related death				1.54(0.64-3.65)	0(-)	0.62(0.15-2.44)
	Obstetric haemorrhage				2.15(1.03-4.46)	3.08(1.66-5.64)	1.23(0.46-3.24)
	Other and unspecified maternal CoD				3.38(1.88-6.02)	1.85(0.83-4.06)	3.08(1.66-5.64)
**External Causes of death**		**18.57(14.95-22.83)**	**14.59(11.36-18.54)**	**16.44(13.03-20.55)**	**6.46(4.24-9.72)**	**4.92(3.03-7.89)**	**6.46(4.24-9.72)**
	Transport Accident	6.10(4.08-9.02)	7.43(5.17-10.56)	7.96(5.61-11.17)	1.54(0.64-3.65)	2.15(1.03-4.46)	2.77(1.44-5.25)
	Accidental poisoning & noxious substance	0.79(0.26-2.45)	0(-)	0(-)	0.62(0.15-2.44)	0(-)	0(-)
	Contact with venomous plant/animal	3.18(1.81-5.53)	0(-)	0(-)	0.31(0.04-2.17)	0(-)	0(-)
	Assault	3.18(1.81-5.53)	3.71(2.21-6.18)	5.57(3.65-8.40)	0.62(0.15-2.44)	1.23(0.46-3.24)	1.23(0.46-3.24)
	Accidental drowning and submersion	0.53(0.13-2.10)	0.53(0.13-2.10)	0.53(0.13-2.10)	0.31(0.04-2.17)	0(-)	0(-)
	Accidental exposure to smoke fire & flame	0.27(0.04-1.87)	0.27(0.04-1.87)	0(-)	0.31(0.04-2.17)	0.31(0.04-2.17)	0(-)
	Intentional self-harm	3.44(2.01-5.86)	1.86(0.89-3.85)	0.53(0.13-2.10)	0.62(0.15-2.44)	0.24(0.03-1.68)	0(-)
	Accidental fall	0.79(0.26-2.45)	0.79(0.26-2.45)	1.86(0.89-3.85)	0.31(0.04-2.17)	1.23(0.46-3.24)	2.46(1.23-4.86)
	Other and unspecified external CoD	3.18(1.81-5.53)	0(-)	0(-)	1.85(0.83-4.06)	0(-)	0(-)
**Undetermined**			**1.59(0.71-3.51)**	**0.79(0.26-2.45)**		**2.15(1.03-4.46)**	**2.77(1.44-5.25)**

**Fig 3:**
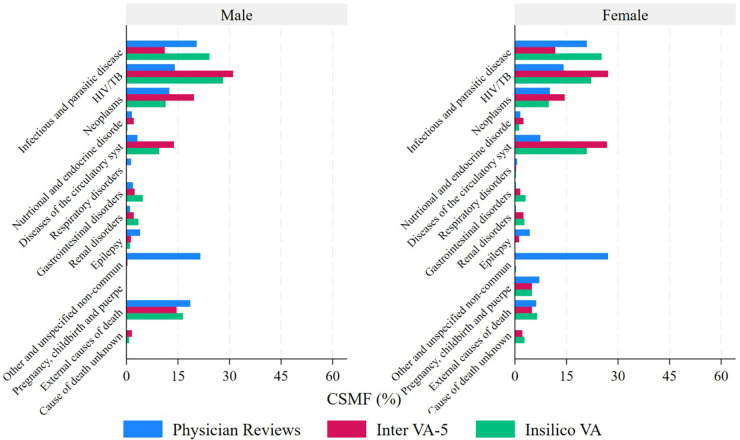
Cause-specific mortality fraction at the individual level comparing computer algorithms and Physician reviews stratified by sex in Rakai (WHO cause-list).

Fig 3 shows a distribution of CSMF at the individual level based on the WHO cause-list of fifteen CoD. Based on the CSMF, while each method had a different order of the leading causes of death, HIV/TB and neoplasms were consistently part of the five leading causes of death for each of the three methods for both males and females. Based on Physician reviews, HIV/TB was the fourth and third leading CoD in males (14.1%) and females (14.2%), respectively, while neoplasms were the fifth and fourth leading CoD in males (12.5%) and females (10.2%), respectively. Based on the InterVA-5 method, HIV/TB was the leading CoD in males (31%) and females (27.2%), respectively, while neoplasms were the second and third leading CoD in males (19.6%) and females (14.6%). Considering the InSilicoVA-v1.4 method HIV/TB was the leading CoD in males (28.1%) and second leading CoD in females (22.3%), respectively, while neoplasms were the fourth leading CoD in males (11.4%) and females (9.9%) (Fig 3).

The percentage agreement with Physician reviews overall (InSilicoVA-v1.4 – 38.2% (95% CI: 34.7-41.8) and InterVA-5 – 40.5% (95% CI:34.7-41.8)) and for sex and age categories was slightly higher for InterVA-5 compared to InSilicoVA-v1.4, except for older people aged 50+ where the percentage agreement with Physician reviews was almost the same at 32% for both methods. Based on the fourteen WHO cause lists, the InSilicoVA-v1.4 had a higher percentage agreement with Physician reviews than the InterVA-5 overall (44.4%, 95% CI:40.8-48.2 Vs 41.0%, 95% CI:37.4-44.7) and for all sex and age categories (Table 4).

**Table 4:** Comparison of InterVA-5 and InSilicoVA against the Physician reviews in Rakai.

	Individual-level agreement			Population-level agreement	
	Overall percentage agreement (95% CI)	Overall percentage agreement (WHO cause list) (95% CI)	Overall percentage agreement (Top cause - WHO cause list) (95% CI)	CSMF - Accuracy (95% CI)	Spearman’s correlation (95% CI)
**Total Sample N = 702**					
InterVA-5	40.46(36.88-44.14)	41.03(37.44-44.71)	12.54(10.28-15.20)	0.88(0.85-0.90)	0.48(0.43-0.54)
InSilico VA	38.18(34.65-41.84)	44.44(40.79-48.15)	11.82(9.63-14.43)	0.83(0.79-0.87)	0.49(0.44-0.55)
**Males n = 377**					
InterVA-5	39.52(34.69-44.57)	44.03(39.08-49.10)	12.99(9.95-16.79)	0.87(0.83-0.91)	0.54(0.46-0.60)
InSilico VA	36.34(31.62-41.34)	45.89(40.90-50.96)	12.20(9.26-15.92)	0.81(0.77–0.86)	0.52(0.45-0.59)
**Females n = 325**					
InterVA-5	41.54(36.28-46.99)	37.54(32.42-42.95)	12.00(8.88-16.02)	0.88(0.84–0.92)	0.42(0.32-0.50)
InSilico VA	40.31(35.09-45.76)	42.77(37.48-48.23)	10.77(7.82-14.65)	0.85(0.79-0.89)	0.45(0.36-0.54)
**15-49 years n = 276**					
InterVA-5	52.19(46.25-58.07)	56.93(50.97-62.69)	25.18(20.38-30.68)	0.86(0.81-0.91)	0.66(0.59-0.72)
InSilico VA	48.18(42.29-54.12)	60.22(54.28-65.87)	22.99(18.37-28.37)	0.84(0.79–0.89)	0.71(0.64-0.76)
**50 + years n = 426**					
InterVA-5	32.94(28.64-37.55)	30.84(26.63-35.39)	6.07(4.16-8.78)	0.88(0.85-0.92)	0.33(0.24-0.41)
InSilico VA	31.78(27.52-36.35)	34.35(29.99-38.99)	11.45(8.75-14.84)	0.82(0.77-0.87)	0.32(0.23-0.40)

At the population level, the CSMF accuracy was above 50% (88%, 95% CI:85–90 InterVA-5 and 83%, 95% CI:79–87 InSilicoVA-v1.4) overall, and above 80% for both algorithms by sex and age categories, lowest for males for InSilicoVA-v1.4 (81%, 95% CI:77–86), suggesting strong agreement with Physician reviews by sex and age categories (Table 4). The Spearman’s rank correlation coefficient was highest for individuals aged 15–49 years at 0.71 (95% CI: 0.64-0.76) for InSilicoVA-v1.4, suggesting a strong positive correlation with physician reviews. On the other hand, the correlation was lowest for individuals aged 50 + years at 0.32 (95% CI: 0.23-0.40) for InSilicoVA-v1.4, suggesting that InSilicoVA-v.14 aligns much less closely with physician reviews for older adults (Table 4). An additional analysis was conducted without applying the 0.4 probability threshold for the algorithms. A slight increase was observed in the Spearman’s correlation coefficient, indicating stronger positive relationships. The results are presented in S4 Table.

**Fig 4:**
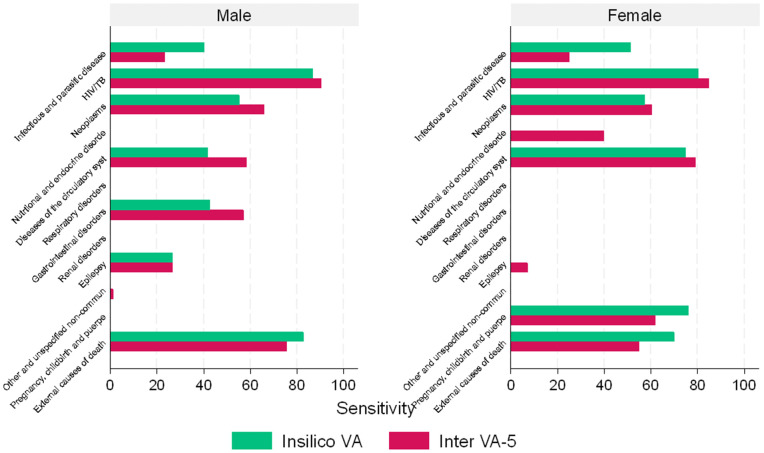
Sensitivity of InSilicoVA-v1.4 and InterVA-5 compared to Physician reviews stratified by sex in Rakai at the population level.

Fig 4 shows the sensitivity results of the methods in Rakai (comparing InSilicoVA-v1.4 and InterVA-5 with Physician reviews). The sensitivity of the InSilicoVA-v1.4 was greater than 50% in females and males, except for infectious diseases, diseases of the circulatory system, epilepsy and gastrointestinal disorders. InSilicoVA-v1.4 was more sensitive (compared to InterVA-5) in classifying deaths to infectious and parasitic diseases (excluding HIV/TB), pregnancy and childbirth causes, and external causes, while the InterVA-5 was more sensitive (compared to InSilicoVA-v1.4) in classifying deaths to non-communicable causes and HIV/TB-related causes, just as the Physician reviews.

### Analysis 2: Comparison of computer algorithms with one another in Rakai and Kyamulibwa

**Fig 5:**
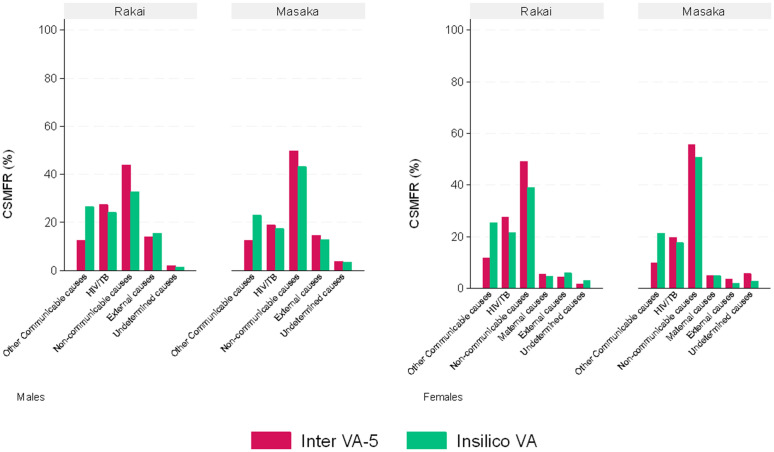
Overall cause-specific mortality fraction at the broad level by computer algorithm stratified by sex in Rakai and Kyamulibwa.

Tables 5 and 6 show variations in the distribution of CoD by each of the algorithms by sex. While the highest number of deaths were assigned to the non-communicable causes of death for both algorithms in the two HDSS, the InSilicoVA-v1.4 reported a slightly lower proportion of deaths assigned to this category (Rakai males - 32.67%) compared to InterVA-5 (Rakai males – 43.92%). In this category, both methods in HDSS showed cardiac disease as the leading cause of death. InterVA-5 assigned a higher proportion of deaths to the HIV/TB-related (Rakai males – 27.22%, Kyamulibwa males – 18.95%) category compared to InSilicoVA-v1.4 in both sites (Rakai males – 23.96%, Kyamulibwa males – 17.54%). Other communicable causes were the third leading cause of death in both sites. While Acute respiratory infections were assigned as the leading CoD in the other communicable causes category, by InterVA-5 in both sites, InSilicoVA-v1.4 reported other and unspecified infectious diseases as the leading CoD in this category in Rakai. Overall, all methods assigned some deaths to an undetermined category (Tables 5 and 6, Fig 5).

**Table 5:** CSMF (%) stratified by the different algorithms in Males in Rakai and Kyamulibwa.

Broad Cause of death	Specific COD	Rakai (N = 551)		Kyamulibwa (N = 285)	
		InterVA-5	InSillico VA	InterVA-5	InSillico VA
		n(n/N: 95% CI)	n(n/N: 95% CI)	n(n/N: 95% CI)	n(n/N: 95% CI)
**Communicable diseases**		**70(12.70: 10.17-15.76)**	**145(26.32: 22.80-30.16)**	**36(12.63: 9.24-17.03)**	**65(22.81:18.29-28.05)**
	Sepsis (non-obstetric)	3(0.54: 0.18-1.68)	0(-)	1(0.35: 0.05-2.46)	0(-)
	Acute resp infect incl pneumonia	32(5.81: 4.13-8.10)	32(5.81: 4.13-8.10)	31(10.88: 7.75-15.06)	37(12.98: 9.55-17.42)
	Diarrhoeal diseases	0(-)	0(-)	1(0.35: 0.05-2.46)	0(-)
	Malaria	1(0.18: 0.02-1.28)	5(0.91: 0.38-2.16)	0(-)	3(1.05: 0.33-3.22)
	Tetanus	0(-)	0(-)	0(-)	1(0.35: 0.05-2.46)
	Meningitis and encephalitis	6(1.09: 0.49-2.40)	8(1.45: 0.73-2.88)	0(-)	0(-)
	Haemorrhagic fever (non-dengue)	0(-)	0(-)	0(-)	1(0.35: 0.05-2.46)
	Dengue fever	0(-)	0(-)	0(-)	0(-)
	Other and unspecified infectious diseases	28(5.08: 3.53-7.26)	100(18.15: 15.15-21.59)	3(1.05: 0.34-3.22)	23(8.07: 5.42-11.86)
**HIV/TB related**		**150(27.22: 23.67-31.09)**	**132(23.96: 20.57-27.70)**	**54(18.95: 14.80-23.93)**	**50(17.54: 13.55-22.42)**
	HIV/AIDS related death	114(20.69: 17.51-24.28)	92(16.7: 13.81-20.05)	36(12.63: 9.24-17.03)	28(9.82: 6.86-13.87)
	Pulmonary tuberculosis	36(6.53: 4.75-8.93)	40(7.26: 5.37-9.75)	18(6.32: 4.01-9.81)	22(7.72: 5.13-11.45)
**Non-communicable diseases**		**242(43.92: 39.82-48.10)**	**180(32.67: 28.87-36.70)**	**142(49.82: 44.03-55.62)**	**123(43.16: 37.51-48.99)**
	Oral neoplasms	2(0.36: 0.09-1.44)	0(-)	0(-)	0(-)
	Digestive neoplasms	61(11.07: 8.71-13.98)	38(6.9: 5.06-9.34)	20(7.02: 4.57-10.64)	17(5.96: 3.74-9.39)
	Respiratory neoplasms	14(2.54: 1.51-4.25)	6(1.09: 0.49-2.40)	12(4.21: 2.40-7.28)	7(2.46: 1.17-5.07)
	Breast neoplasms	0(-)	0(-)	0(-)	0(-)
	Reproductive neoplasms MF	1(0.18: 0.03-1.28)	0(-)	5(1.75: 0.73-4.15)	4(1.4: 0.53-3.69)
	Other and unspecified neoplasms	23(4.17: 2.79-6.21)	13(2.36: 1.37-4.02)	6(2.11: 0.95-4.61)	9(3.16: 1.65-5.97)
	Severe malnutrition	0(-)	0(-)	1(0.35: 0.05-2.46)	0(-)
	Diabetes mellitus	12(2.18: 1.24-3.79)	2(0.36: 0.09-1.44)	12(4.21: 2.40-7.28)	9(3.16: 1.65-5.97)
	Acute cardiac disease	9(1.63: 0.85-3.11)	3(0.54: 0.18-1.68)	14(4.91: 2.93-8.13)	10(3.51: 1.89-6.41)
	Stroke	54(9.8: 7.58-12.58)	41(7.44: 5.52-9.95)	19(6.67: 4.29-10.22)	18(6.32: 4.01-9,81)
	Other and unspecified cardiac disease	25(4.54: 3.08-6.63)	21(3.81: 2.49-5.78)	8(2.81: 1.41-5.52)	2(0.7: 0.18-2.77)
	Chronic obstructive pulmonary disease	0(-)	0(-)	3(1.05: 0.34-3.22)	0(-)
	Asthma	0(-)	0(-)	0(-)	0(-)
	Acute abdomen	3(0.54: 0.18-1.68)	15(2.72: 1.65-4.47)	15(5.26: 3.19-8.55)	19(6.67: 4.29-10.22)
	Liver cirrhosis	10(1.81: 0.98-3.34)	10(1.81: 0.98-3.34)	12(4.21: 2.40-7.28)	10(3.51: 1.89-6.41)
	Renal failure	11(2: 1.11-3.57)	17(3.09: 1.93-4.91)	5(1.75: 0.73-4.15)	3(1.05: 0.34-3.22)
	Epilepsy	16(2.9: 1.79-4.69)	14(2.54: 1.51-4.25)	4(1.4: 0.53-3.69)	2(0.7: 0.18-2.77)
	Other and unspecified NCD	1(0.18: 0.03-1.28)	0(-)	6(2.11: 0.95-4.61)	13(4.56: 2.66-7.70)
**External causes**		**78(14.16: 11.48-17.33)**	**86(15.61: 12.81-18.89)**	**42(14.74: 11.0719.35)**	**37(12.98: 9.55-17.42)**
	Road traffic accident	38(6.9: 5.06-9.34)	36(6.53: 4.75-8.93)	15(5.26: 3.19-8.55)	11(3.86: 2.15-6.84)
	Other transport accident	0(-)	2(0.36: 0.09-1.44)	0(-)	1(0.35: 0.05-2.46)
	Accid fall	6(1.09: 0.49-2.40)	12(2.18: 1.24-3.79)	4(1.4: 0.53-3.69)	5(1.75: 0.73-4.15)
	Accid drowning and submersion	2(0.36: 0.09-1.44)	4(0.73: 0.27-1.92)	1(0.35: 0.05-2.46)	1(0.35: 0.05-2.46)
	Accid expos to smoke fire & flame	2(0.36: 0.09-1.44)	0(-)	1(0.35: 0.05-2.46)	0(-)
	Intentional self-harm	10(1.81: 0.98-3.34)	2(0.36: 0.09-1.44)	5(1.75: 0.73-4.15)	1(0.35: 0.05-2.46)
	Assault	20(3.63: 2.35-5.56)	30(5.44: 3.83-7.68)	14(4.91: 2.93-8.13)	18(6.32: 4.01-9.81)
	Exposure to force of nature	0(-)	0(-)	0(-)	0(-)
	Other and unspecified external CoD	0(-)	0(-)	2(0.7: 0.18-2.77)	0(-)
**Undetermined causes**		**11(2.00: 1.11-3.57)**	**8(1.45: 0.72-2.88)**	**11(3.86: 21.47-6.84)**	**10(3.51: 1.89-6.41)**

**Table 6:** CSMF stratified by the different algorithms in Females in Rakai and Kyamulibwa.

		Rakai (N = 486)		Kyamulibwa (N = 242)	
		InterVA-5	InSillico VA	InterVA-5	InSillico VA
		n(n/N: 95% CI)	n(n/N: 95% CI)	n(n/N: 95% CI)	n(n/N: 95% CI)
**Communicable diseases**		**57(11.73: 9.15-14.91)**	**123(25.31: 21.64-29.37)**	**24(9.92: 6.7-14.38)**	**52(21.49: 16.75-27.12)**
	Sepsis (non-obstetric)	2(0.41: 0.10-1.63)	0(-)	2(0.83: 0.21-3.25)	0(-)
	Acute resp infect incl pneumonia	30(6.17:4.35-8.69)	41(8.44: 6.27-11.26)	14(5.79: 3.45-9.54)	27(11.16: 7.76-15.79)
	Diarrhoeal diseases	1(0.21: 0.03-1.45)	1(0.21: 0.03-1.45)	2(0.83: 0.21-3.25)	1(0.41: 0.06-2.89)
	Malaria	0(-)	5(1.03: 0.43-2.45)	1(0.41: 0.06-2.89)	6(2.48: 1.11-5.42)
	Tetanus	0(-)	0(-)	0(-)	0(-)
	Meningitis and encephalitis	10(2.06: 1.11-3.78)	2(0.41: 0.10-1.63)	2(0.83: 0.21-3.25)	0(-)
	Haemorrhagic fever (non-dengue)	0(-)	0(-)	0(-)	0(-)
	Dengue fever	0(-)	0(-)	0(-)	0(-)
	Other and unspecified infectious diseases	14(2.88: 1.71-4.81)	74(15.23: 12.29-18.71)	3(1.24: 0.39-3.78)	18(7.44: 4.73-11.51)
**HIV/TB related**		**133(27.37: 23.58-31.51)**	**106(21.81: 18.36-25.71)**	**48(19.83: 15.27-25.35)**	**43(17.77: 13.44-23.12)**
	HIV/AIDS related death	128(26.34: 22.61-30.44)	97(19.96: 16.64-23.75)	40(16.53: 12.35-21.77)	35(14.46: 10.56-19.49)
	Pulmonary tuberculosis	5(1.03: 0.43-2.45)	9(1.85: 0.97-3.52)	8(3.31: 1.66-6.58)	8(3.31:16.59-6.48)
**Non-communicable diseases**		**239(49.18: 44.75-53.62)**	**190(39.09: 34.85-43.51)**	**135(55.79:49.45-61.93)**	**123(50.83: 44.53-57.09)**
	Oral neoplasms	0(-)	0(-)	3(1.24: 0.39-3.78)	2(0.83: 0.21-3.25)
	Digestive neoplasms	28(5.76: 4.01-8.22)	23(4.73: 3.16-7.02)	7(2.89: 1.38-5.95)	6(2.48: 1.12-5.42)
	Respiratory neoplasms	2(0.41: 0.10-1.63)	0(-)	11(4.55: 2.53-8.03)	8(3.31: 1.66-6.48)
	Breast neoplasms	3(0.62: 0.19-1.89)	3(0.62: 0.19-1.89)	1(0.41: 0.06-2.89)	0(-)
	Reproductive neoplasms MF	28(5.76:4.00-8.22)	13(2.67: 1.56-4.55)	15(6.2: 3.77-10.04)	9(3.72: 1.94-7.00)
	Other and unspecified neoplasms	8(1.65: 0.82-3.26)	6(1.23: 0.56-2.72)	9(3.72: 1.94-7.00)	13(5.37: 3.14-9.04)
	Severe malnutrition	0(-)	1(0.21: 0.03-1.45)	1(0.41: 0.06-2.89)	2(0.83: 0.21-3.25)
	Diabetes mellitus	16(3.29: 2.02-5.31)	7(1.44: 6.88-2.99)	10(4.13: 2.23-7.52)	5(2.07: 0.86-4.88)
	Acute cardiac disease	11(2.26: 1.26-4.04)	0(-)	12(4.96: 2.83-8.54)	7(2.89: 1.38-5.95)
	Stroke	69(14.2: 11.36-17.59)	67(13.79: 10.99-17.15)	17(7.02: 4.41-11.02)	12(4.96:2.83-8.54)
	Other and unspecified cardiac disease	36(7.41: 5.39-10.10)	29(5.97: 4.18-8.46)	22(9.09: 6.05-13.43)	19(7.85: 5.06-11.99)
	Chronic obstructive pulmonary disease	1(0.21: 0.03-1.45)	1(0.21: 0.03-1.45)	1(0.41: 0.06-2.89)	1(0.41: 0.06-2.89)
	Asthma	1(0.21: 0.03-1.45)	0(-)	1(0.41: 0.06-2.89)	1(0.41: 0.06-2.89)
	Acute abdomen	6(1.23: 0.56-2.72)	13(2.67: 1.56-4.55)	10(4.13: 2.23-7.52)	17(7.02: 4.41-11.02)
	Liver cirrhosis	7(1.44: 0.69-2.99)	7(1.44: 0.69-2.99)	8(3.31: 1.66-6.48)	4(1.65: 0.62-4.33)
	Renal failure	12(2.47: 1.41-4.29)	18(3.7: 2.34-5.80)	2(0.83: 0.21-3.25)	0(-)
	Epilepsy	11(2.26:1.26-4.04)	1(0.21: 0.03-1.45)	1(0.41: 0.06-2.89)	1(0.41: 0.06-2.89)
	Other and unspecified NCD	0(-)	1(0.21: 0.03-1.45)	4(1.65: 0.62-4.33)	16(6.61: 4.08-10.53
**Maternal Causes**		**27(5.56: 3.83-7.98)**	**23(4.73: 3.16-7.02)**	**12(4.96: 2.83-8.54)**	**12(4.96: 2.83-8.54)**
	Abortion-related death	0(-)	2(0.41: 0.10-1.63)	0(-)	2(0.83: 0.21-3.25)
	Pregnancy-induced hypertension	10(2.06: 1.11-3.78)	7(1.44: 0.69-2.99)	0(-)	0(-)
	Obstetric haemorrhage	14(2.88: 1.71-4.81)	4(0.82: 0.31-2.17)	8(3.31: 1.67-6.48)	1(0.41: 0.06-2.89)
	Obstructed labour	0(-)	0(-)	0(-)	1(0.41: 0.06-2.89)
	Pregnancy-related sepsis	2(0.41: 0.10-1.63)	1(0.21: 0.03-1.45)	1(0.41: 0.06-2.89)	3(1.24: 0.39-3.78)
	Anaemia of pregnancy	0(-)	1(0.21: 0.03-1.45)	0(-)	1(0.41: 0.06-2.89)
	Ruptured uterus	1(0.21: 0.03-1.44)	8(1.65: 0.08-3.26)	2(0.83: 0.21-3.25)	4(1.65: 0.62-4.33)
	Other and unspecified maternal CoD	0(-)	0(-)	1(0.41: 0.06-2.89)	0(-)
**External causes**		**21(4.32: 2.83-6.54)**	**29(5.97: 4.18-8.46)**	**9(3.72: 1.94-7.00)**	**5(2.07: 0.86-4.88)**
	Road traffic accident	8(1.65: 0.82-3.26)	8(1.65: 0.82-3.26)	2(0.83: 0.21-3.25)	1(0.41: 0.06-2.89)
	Other transport accident	0(-)	3(0.62: 0.19-1.89)	0(-)	0(-)
	Accid fall	6(1.23: 0.56-2.72)	13(2.67: 1.56-4.55)	0(-)	2(0.83: 0.21-3.25)
	Accid drowning and submersion	0(-)	0(-)	0(-)	0(-)
	Accid expos to smoke fire & flame	1(0.21: 0.03-1.45)	0(-)	1(0.41: 0.06-2.89)	0(-)
	Intentional self-harm	1(0.21: 0.03-1.45)	0(-)	3(1.24: 0.39-3.78)	0(-)
	Assault	5(1.03: 0.43-2.45)	5(1.03: 0.43-2.45)	1(0.41: 0.06-2.89)	1(0.41: 0.06-2.89)
	Exposure to force of nature	0(-)	0(-)	0(-)	1(0.41: 0.06-2.89)
	Other and unspecified external CoD	0(-)	0(-)	2(0.83: 0.21-3.25)	0(-)
**Undetermined causes**		**9(1.85: 0.97-3.52)**	**15(3.09: 1.87-5.06)**	**14(5.79: 3.45-9.54)**	**7(2.89: 1.38-5.95)**

Table 7 shows the algorithms’ percentage agreement in assigning causes of death at the individual level and population, stratified by age and sex. For both HDSS, the percentage agreement between InterVA-5 and InSilicoVA-v1.4 was greater than 60% for both males and females across all age groups except females aged 15–29 (Rakai females - 52.54% and Kyamulibwa females—42.11%).

**Table 7:** Comparison of Percentage agreement between computer algorithms by age and sex in Rakai and Kyamulibwa.

Population Level agreement	Rakai (n/N, %)		Kyamulibwa (n/N, %)	
	Males	Females	Males	Females
Overall % agreement	440/551(79.85)	374/486(76.95)	236/285(82.81)	189/242(78.10)
15-29	59/67(88.06)	37/59(62.71)	20/27(74.07)	14/19(73.68)
30-49	146/176(82.95)	93/109(85.32)	37/45(82.22)	29/37(78.38)
50+	235/308(76.30)	244/318(76.73)	179/213(84.04)	147/186(79.03)
**Individual level agreement**				
Overall % agreement	372/551(67.51)	318/486(65.43)	205/285(71.93)	151/242(62.40)
15-29	53/67(79.10)	31/59(52.54)	15/27(55.56)	8/19(42.11)
30-49	124/176(70.45)	80/109(73.39)	36/45(80.00)	24/37(64.66)
50+	195/308(63.31)	207/318(65.09)	154/213(72.30)	119/186(63.98)

## Discussion

Findings from this study suggest that, while commonly implemented computer algorithms achieved moderate agreement with physician reviews (around 60%) and slightly agree with each other in identifying broad causes of death in this population-based study, there remains room for improvement. For broad CoD there was more than 60% agreement in the proportion allocated to each cause, the CSMF, but for individual deaths, there was a much lower agreement. For the WHO CoD and the Harmonised CoD, with more categories, there was much lower agreement between physician reviews and the computer algorithms.

For all methods, communicable causes of death (HIV/TB plus other communicable causes of death) were the leading causes of death. Non-communicable diseases had a high CSMF ranging from 30% to 51% across all methods. The moderate agreement in the proportion of deaths due to communicable and non-communicable causes showed that the computer algorithms have good agreement on the CoD distributions at the population level. Performance agreement was better at the population level because it is easier to leverage population-level data to identify common causes of death based on general patterns that apply to many individuals. On the other hand, at the individual level, performance agreement was poor because physicians bring a high level of expertise, clinical experience, and the ability to evaluate nuanced patient histories, comorbidities, and other factors that are difficult for algorithms to capture.

Physicians can draw on qualitative information from the narrative section of the verbal autopsy. In contrast, the current versions of InSilicoVA-v1.4 and InterVA-5 do not incorporate this narrative information because it is not captured as structured signs or symptoms within their algorithms. Finding ways to include the free-text narratives in the development of the algorithms may improve the completeness in reporting on the symptoms information of the InterVA-5 and InSilicoVA-v1.4. This addition would improve the individual accuracy in predicting CoD and would be beneficial before replacing the Physician reviews with computer algorithms in assigning individual CoD.

Further, sensitivity analyses at an individual level showed that the performance agreement of both algorithms was more than 50% for all causes except infectious and parasitic diseases (excluding HIV/TB) in all subgroups. The poor performance of the algorithms in identifying infectious diseases (excluding HIV/TB) at the individual level could be because of the overlapping signs and symptoms. Distinguishing similar signs and symptoms may be context-dependent (like prior medical history and living conditions), which may not be coded for inclusion in the algorithms and analysis used by InterVA-5. This low sensitivity underscores the challenges of the InterVA-5 in classifying other communicable causes of death, hence, further investigations are required to inform the revision and development of newer versions of the InterVA-5. However, InSilicoVA-v1.4 showed a higher sensitivity in identifying infectious diseases (excluding HIV/TB), partially because it uses more advanced machine learning or statistical models compared to InterVA-5. These findings are comparable to those documented by other studies regarding the performance of computer algorithms versus physician reviews. Byass et al. [21] and Groenewald et al. [28] showed that InterVA-5 performed well at detecting HIV/TB-related deaths, which is similar to findings in this study, where InterVA-5 had a sensitivity of 80% in identifying HIV/TB-related deaths.

The findings of this study showed that the CSMF accuracy in assigning the cause of death between the InSilicoVA-v1.4 and InterVA-5 methods at the population level was more than 75%, which is almost similar to the findings of McKormick et al. [19]. In this study, InterVA-5 achieved a CSMF accuracy rate exceeding 80% when compared with physician reviews among individuals aged 50 years and older. This finding is consistent with previous reports by Jha et al. [29] (approximately 80%) and Groenewald et al. (84%) [28]. Our findings further reveal a divergence between CSMF accuracy and Spearman’s rank correlation: the algorithms reproduced population-level cause distributions well but aligned less consistently with physician rankings. Similar patterns have been reported by other studies [19,25,30], highlighting that automated tools like InterVA and InSilicoVA achieve robust proportional accuracy. These results underscore that while algorithms are valuable for population-level surveillance, physician review remains essential for resolving individual-level uncertainties and refining cause attribution [14,31].

A key limitation of this study is the use of physician review (PCVA) as the gold standard. Although PCVA has been widely employed as a reference method in verbal autopsy research [32], it is subject to several important limitations. These include its imperfect agreement with causes of death established through complete diagnostic autopsy or minimally invasive tissue sampling [33]. Other limitations include inter-reviewer variability, reliance on subjective clinical judgment, and well-documented misclassification errors [32,34]. Such errors are particularly pronounced for causes of death with overlapping or non-specific symptomatology—for example, HIV/AIDS-related deaths are frequently misclassified as tuberculosis or other respiratory infections, while malaria may be confused with acute febrile illnesses such as sepsis or meningitis [35,36]. This diagnostic ambiguity undermines the reliability of PCVA at the individual level and introduces bias into cause-specific mortality fractions, particularly in contexts with limited access to clinical records or diagnostic testing [34,37]. Although distinguishing specific pathogens (for example, viral versus bacterial pneumonia) would provide more precise guidance for clinical management and vaccine policy, verbal autopsy methods are not designed to reliably capture this level of diagnostic detail. The information obtained from interviews with caregivers inherently limits the precision of cause-of-death attribution and attempts to make finer distinctions can result in substantial misclassification. For this reason, broader syndromic categories are generally recommended to maintain accuracy, consistency, and comparability across settings. More detailed, pathogen-specific studies are needed to complement VA-based mortality estimates. In addition, this study used data collected with the WHO 2012 verbal autopsy instrument. Two updated versions have since been released with revised questions and cause-of-death classifications. Further studies using data collected with the newer versions are needed to assess algorithm performance with the most current tools.

Although verbal autopsy algorithms have been evaluated previously, this study introduces several important advances. We use a rigorously characterised, population-based dataset with physician-reviewed causes of death as the reference standard and apply a standardised comparison framework across multiple performance metrics. To our knowledge, this is the first evaluation focused on adult deaths within a community-based study in Uganda, generating context-specific evidence to inform the implementation of automated cause-of-death assignment in real-world surveillance systems. A key strength of this work is the direct comparison of two automated verbal autopsy algorithms against physician review using population-based data, allowing for detailed cause-specific performance assessment relevant to public health surveillance in low-resource settings. While agreement between methods was moderate and performance varied by cause of death, these findings provide critical insight into algorithm applicability and highlight the need for further validation across diverse settings and larger datasets to strengthen generalisability and practical utility.

The findings of this study have important implications for health policy, particularly in rural regions where mortality data remain sparse and civil registration systems are limited. The consistency and agreement in estimating population-level cause of death distributions by the computer-based VA algorithms make them valuable tools for informing public health strategies in resource-limited settings, where comprehensive data collection is often infeasible [25,31,38]. Policymakers may consider a tiered surveillance approach—utilising automated VA tools for routine mortality monitoring while reserving physician review for select or high-priority cases [30]. Additionally, locally calibrating algorithmic models using region-specific data can improve contextual accuracy and reduce misclassification [39].

## Conclusion

While computer algorithms may not capture the nuance of physician-coded deaths for individual cases, they offer a practical and scalable tool that can support mortality surveillance in resource-limited settings. However, given the modest performance observed, these algorithms should be viewed as complementary to—rather than replacements for—physician review. Substantial improvements, including better integration of narrative information and continued refinement of symptom–cause models, are needed before they can reliably guide policy or programmatic decisions. Strengthening health systems, diagnostic capacity, and training will also remain essential components of any comprehensive mortality surveillance strategy.

## Supporting information

S1 Table Harmonization of the causes of death assigned by Physician reviews, InterVA-5, and InSilicoVA. (DOCX)

S2 Table Classification of undetermined Physician CoD (N = 236) by the algorithms. (DOCX)

S3 Table A mapping of the ICD-10 codes to the WHO CoD list and Broad CoD list. (DOCX)

S4 Table Comparison of InterVA-5 and InSilicoVA with Physician Reviews in Rakai, without Application of the 0.4 Cutoff on probability of CoD assigned by the algorithms. (DOCX)

## References

[1] Nations U. Transforming our world: The 2030 agenda for sustainable development. New York: United Nations, Department of Economic and Social Affairs; 2015.

[2] Adair T. Who dies where? Estimating the percentage of deaths that occur at home. BMJ Glob Health. 2021;6(9):e006766. doi: 10.1136/bmjgh-2021-006766 34479953 PMC8420738

[3] Jha P. Reliable direct measurement of causes of death in low- and middle-income countries. BMC Med. 2014;12:19. doi: 10.1186/1741-7015-12-19 24495839 PMC3912491

[4] D’Ambruoso L, Boerma T, Byass P, Fottrell E, Herbst K, Källander K, et al. The case for verbal autopsy in health systems strengthening. Lancet Glob Health. 2017;5(1):e20–1. doi: 10.1016/S2214-109X(16)30332-1 27866775

[5] WHO. Verbal Autopsy Standards: 2012 WHO Verbal Autopsy Instrument. 2012.

[6] WHO. International statistical classification of diseases and related health problems - 10th revision (ICD-10). 2016.

[7] Murray CJ, Lopez AD, Shibuya K, Lozano R. Verbal autopsy: advancing science, facilitating application. Popul Health Metr. 2011;9:18. doi: 10.1186/1478-7954-9-18 21794169 PMC3160911

[8] Nichols EK, Byass P, Chandramohan D, Clark SJ, Flaxman AD, Jakob R, et al. The WHO 2016 verbal autopsy instrument: An international standard suitable for automated analysis by InterVA, InSilicoVA, and Tariff 2.0. PLoS Med. 2018;15(1):e1002486. doi: 10.1371/journal.pmed.1002486 29320495 PMC5761828

[9] Flaxman AD, Vahdatpour A, James SL, Birnbaum JK, Murray CJ, Population Health Metrics Research Consortium (PHMRC). Direct estimation of cause-specific mortality fractions from verbal autopsies: multisite validation study using clinical diagnostic gold standards. Popul Health Metr. 2011;9:35. doi: 10.1186/1478-7954-9-35 21816098 PMC3160928

[10] James SL, Flaxman AD, Murray CJ, Population Health Metrics Research Consortium (PHMRC). Performance of the Tariff Method: validation of a simple additive algorithm for analysis of verbal autopsies. Popul Health Metr. 2011;9:31. doi: 10.1186/1478-7954-9-31 21816107 PMC3160924

[11] Desai N, Aleksandrowicz L, Miasnikof P, Lu Y, Leitao J, Byass P, et al. Performance of four computer-coded verbal autopsy methods for cause of death assignment compared with physician coding on 24,000 deaths in low- and middle-income countries. BMC Med. 2014;12:20. doi: 10.1186/1741-7015-12-20 24495855 PMC3912488

[12] Mpimbaza A, Filler S, Katureebe A, Quick L, Chandramohan D, Staedke SG. Verbal Autopsy: Evaluation of Methods to Certify Causes of Death in Uganda. PLoS One. 2015;10(6):e0128801. doi: 10.1371/journal.pone.0128801 26086600 PMC4472780

[13] Pham BN, Abori N, Maraga S, Jorry R, Jaukae GS, Silas VD, et al. Validating the InterVA-5 cause of death analytical tool: using mortality data from the Comprehensive Health and Epidemiological Surveillance System in Papua New Guinea. BMJ Open. 2023;13(5):e066560. doi: 10.1136/bmjopen-2022-066560 37217264 PMC10230342

[14] Flaxman AD, Joseph JC, Murray CJL, Riley ID, Lopez AD. Performance of InSilicoVA for assigning causes of death to verbal autopsies: multisite validation study using clinical diagnostic gold standards. BMC Med. 2018;16(1):56. doi: 10.1186/s12916-018-1039-1 29669548 PMC5907465

[15] Reniers G, Wamukoya M, Urassa M, Nyaguara A, Nakiyingi-Miiro J, Lutalo T, et al. Data Resource Profile: Network for Analysing Longitudinal Population-based HIV/AIDS data on Africa (ALPHA Network). Int J Epidemiol. 2016;45(1):83–93. doi: 10.1093/ije/dyv343 26968480 PMC5823235

[16] Asiki G, Murphy G, Nakiyingi-Miiro J, Seeley J, Nsubuga RN, Karabarinde A, et al. The general population cohort in rural south-western Uganda: a platform for communicable and non-communicable disease studies. Int J Epidemiol. 2013;42(1):129–41. doi: 10.1093/ije/dys234 23364209 PMC3600628

[17] Wawer MJ, Gray RH, Sewankambo NK, Serwadda D, Paxton L, Berkley S, et al. A randomized, community trial of intensive sexually transmitted disease control for AIDS prevention, Rakai, Uganda. AIDS. 1998;12(10):1211–25. doi: 10.1097/00002030-199810000-00014 9677171

[18] Byass P, Hussain-Alkhateeb L, D’Ambruoso L, Clark S, Davies J, Fottrell E, et al. An integrated approach to processing WHO-2016 verbal autopsy data: the InterVA-5 model. BMC Med. 2019;17(1):102. doi: 10.1186/s12916-019-1333-6 31146736 PMC6543589

[19] McCormick TH, Li ZR, Calvert C, Crampin AC, Kahn K, Clark SJ. Probabilistic Cause-of-death Assignment using Verbal Autopsies. J Am Stat Assoc. 2016;111(515):1036–49. doi: 10.1080/01621459.2016.1152191 27990036 PMC5154628

[20] Nabukalu D, Calazans JA, Marston M, Calvert C, Nakawooya H, Nansereko B, et al. Estimation of cause-specific mortality in Rakai, Uganda, using verbal autopsy 1999-2019. Glob Health Action. 2024;17(1):2338635. doi: 10.1080/16549716.2024.2338635 38717826 PMC11080674

[21] Byass P, Calvert C, Miiro-Nakiyingi J, Lutalo T, Michael D, Crampin A, et al. InterVA-4 as a public health tool for measuring HIV/AIDS mortality: a validation study from five African countries. Glob Health Action. 2013;6:22448. doi: 10.3402/gha.v6i0.22448 24138838 PMC3800746

[22] Li ZR, Clark S. openVA: automated method for verbal autopsy. 2021. https://cran.r-project.org/package/openVA

[23] Thomas J, L Z, Byass P, McCormick TH, Boyas M, Clark S. InterVA-5: replicate and analyse ‘InterVA-5’. 2021. https://cran.r-project.org/package=InterVA-5

[24] Li ZR, Clark S. InSilicoVA: probabilistic verbal autopsy coding with ‘InSilicova’ algorithm. 2021. https://cran.r-project.org/package=InSilicoVA

[25] Byass P, Chandramohan D, Clark SJ, D’Ambruoso L, Fottrell E, Graham WJ, et al. Strengthening standardised interpretation of verbal autopsy data: the new InterVA-4 tool. Glob Health Action. 2012;5:1–8. doi: 10.3402/gha.v5i0.19281 22944365 PMC3433652

[26] Byass P, Herbst K, Fottrell E, Ali MM, Odhiambo F, Amek N, et al. Comparing verbal autopsy cause of death findings as determined by physician coding and probabilistic modelling: a public health analysis of 54 000 deaths in Africa and Asia. J Glob Health. 2015;5(1):010402. doi: 10.7189/jogh.05.010402 25734004 PMC4337147

[27] StataCorp, Stata Statistical Software: Release 18. College Station, TX: StataCorp LLC; 2023.

[28] Groenewald P, Thomas J, Clark SJ, Morof D, Joubert JD, Kabudula C, et al. Agreement between cause of death assignment by computer-coded verbal autopsy methods and physician coding of verbal autopsy interviews in South Africa. Glob Health Action. 2023;16(1):2285105. doi: 10.1080/16549716.2023.2285105 38038664 PMC10795603

[29] Jha P, Kumar D, Dikshit R, Budukh A, Begum R, Sati P, et al. Automated versus physician assignment of cause of death for verbal autopsies: randomized trial of 9374 deaths in 117 villages in India. BMC Med. 2019;17(1):116. doi: 10.1186/s12916-019-1353-2 31242925 PMC6595581

[30] Murray CJ, Lopez AD, Black R, Ahuja R, Ali SM, Baqui A, et al. Population Health Metrics Research Consortium gold standard verbal autopsy validation study: design, implementation, and development of analysis datasets. Popul Health Metr. 2011;9:27. doi: 10.1186/1478-7954-9-27 21816095 PMC3160920

[31] Serina P, Riley I, Stewart A, James SL, Flaxman AD, Lozano R, et al. Improving performance of the Tariff Method for assigning causes of death to verbal autopsies. BMC Med. 2015;13:291. doi: 10.1186/s12916-015-0527-9 26644140 PMC4672473

[32] Murray CJL, Lozano R, Flaxman AD, Serina P, Phillips D, Stewart A, et al. Using verbal autopsy to measure causes of death: the comparative performance of existing methods. BMC Med. 2014;12:5. doi: 10.1186/1741-7015-12-5 24405531 PMC3891983

[33] Lozano R, Lopez AD, Atkinson C, Naghavi M, Flaxman AD, Murray CJ, et al. Performance of physician-certified verbal autopsies: multisite validation study using clinical diagnostic gold standards. Popul Health Metr. 2011;9:32. doi: 10.1186/1478-7954-9-32 21816104 PMC3160925

[34] Chandramohan D, Setel P, Quigley M. Effect of misclassification of causes of death in verbal autopsy: can it be adjusted? Int J Epidemiol. 2001;30(3):509–14. doi: 10.1093/ije/30.3.509 11416073

[35] Leitao J, Desai N, Aleksandrowicz L, Byass P, Miasnikof P, Tollman S, et al. Comparison of physician-certified verbal autopsy with computer-coded verbal autopsy for cause of death assignment in hospitalized patients in low- and middle-income countries: systematic review. BMC Med. 2014;12:22. doi: 10.1186/1741-7015-12-22 24495312 PMC3912516

[36] Serina P, Riley I, Stewart A, Flaxman AD, Lozano R, Mooney MD, et al. A shortened verbal autopsy instrument for use in routine mortality surveillance systems. BMC Med. 2015;13:302. doi: 10.1186/s12916-015-0528-8 26670275 PMC4681088

[37] Serina P, Riley I, Hernandez B, Flaxman AD, Praveen D, Tallo V, et al. The paradox of verbal autopsy in cause of death assignment: symptom question unreliability but predictive accuracy. Popul Health Metr. 2016;14:41. doi: 10.1186/s12963-016-0104-2 27833460 PMC5101673

[38] Setel PW, Macfarlane SB, Szreter S, Mikkelsen L, Jha P, Stout S, et al. A scandal of invisibility: making everyone count by counting everyone. Lancet. 2007;370(9598):1569–77. doi: 10.1016/S0140-6736(07)61307-5 17992727

[39] Leitao J, Chandramohan D, Byass P, Jakob R, Bundhamcharoen K, Choprapawon C, et al. Revising the WHO verbal autopsy instrument to facilitate routine cause-of-death monitoring. Glob Health Action. 2013;6:21518. doi: 10.3402/gha.v6i0.21518 24041439 PMC3774013

