# Supplementary material for: A comparison of verbal autopsy assignment methods to obtain adult cause-specific mortality in two longitudinal studies in Rakai and Kalungu districts of South Central, Uganda
Source: PLOS Glob Public Health. 2026 Apr 6;6(4):e0006223. doi: 10.1371/journal.pgph.0006223 (PMC13052855; doi:10.1371/journal.pgph.0006223)
Supplement: S1 Table — (DOCX) [file pgph.0006223.s001.docx]

**S1 Table: Harmonisation of the causes of death assigned by Physician reviews, Interva-4, and InSilicoVA**

| **Broad Cause** | **Harmonised cause** | **Physician reviews** | **InterVA-4** | **InSilicoVA** |
| --- | --- | --- | --- | --- |
| *Other and Unspecified communicable causes* | Acute respiratory infection, including pneumonia | Bacterial pneumonia, not elsewhere classified | Acute respiratory infection, including pneumonia | Acute respiratory infection, including pneumonia |
| 1 |  | Pneumonia |  |  |
|  |  | Pneumonia in diseases classified elsewhere |  |  |
|  |  | Pneumonia, organism unspecified |  |  |
|  |  | Viral pneumonia, not elsewhere classified |  |  |
|  |  | Simple and mucopurulent chronic bronchitis |  |  |
|  |  | Pneumonitis due to solids and liquids |  |  |
| 2 | Sepsis (non-obstetric) | Other sepsis | Sepsis (non-obstetric) |  |
| 3 | Diarrhoeal diseases | Diarrhoeal diseases | Diarrhoeal diseases | Diarrhoeal diseases |
|  |  | All other specified diarrhoeal diseases |  |  |
| 4 | Malaria | Malaria | Malaria | Malaria |
|  |  | Other parasitologically confirmed malaria |  |  |
|  |  | Plasmodium falciparum malaria |  |  |
|  |  | Unspecified malaria |  |  |
| 5 | Meningitis and encephalitis | Meningitis | Meningitis and encephalitis | Meningitis and encephalitis |
|  |  | Meningitis due to other and unspecified causes |  |  |
|  |  | Bacterial meningitis, not elsewhere classified |  |  |
|  |  | Meningitis in other infectious and parasitic diseases classified elsewhere |  |  |
| 6 | Other and unspecified infectious diseas | Measles | Haemorrhagic fever (non-dengue) | Haemorrhagic fever (non-dengue) |
|  |  | Acute upper respiratory infections of multiple and unspecified sites | Other and unspecified infectious diseas | Other and unspecified infectious diseas |
|  |  | All other specified Acute Respiratory Infections |  |  |
|  |  | All other specified communicable diseases |  |  |
|  |  | Disorders of peritoneum in infectious diseases classified elsewhere |  |  |
|  |  | Influenza, virus not identified |  |  |
|  |  | Tetanus |  |  |
|  |  | Other tetanus |  |  |
|  |  | Cholera |  |  |
|  |  | Unspecified Acute Respiratory Infections |  |  |
|  |  | Other Salmonella infections |  |  |
|  |  | Unspecified acute lower respiratory infection |  |  |
|  |  | Acute tonsillitis |  |  |
|  |  | Cellulitis |  |  |
|  |  | Cryptococcosis |  |  |
|  |  | Fever of other and unknown origin |  |  |
|  |  | Other mycoses, not elsewhere classified |  |  |
|  |  | Peritonitis |  |  |
|  |  | Relapsing fevers |  |  |
|  |  | Salpingitis and oophoritis |  |  |
|  |  | Shigellosis |  |  |
|  |  | Staphylococcal scalded skin syndrome |  |  |
|  |  | Toxoplasmosis |  |  |
|  |  | Unspecified communicable diseases |  |  |
|  |  | Haemorrhage from respiratory passages |  |  |
|  |  | Encephalitis, myelitis and encephalomyelitis |  |  |
|  |  | All other specified acute febrile illness |  |  |
|  |  | Unspecified Acute febrile illness |  |  |
|  |  | Rabies |  |  |
|  |  | Typhoid and paratyphoid fevers |  |  |
|  |  | Other gastroenteritis and colitis of infectious and unspecified origin |  |  |
| HIV/TB related causes | HIV/AIDS/TB | AIDS | HIV/AIDS related death | HIV/AIDS related death |
|  | 7 | AIDS and Pulmonary tuberculosis | Pulmonary tuberculosis | Pulmonary tuberculosis |
|  |  | Human immunodeficiency virus [HIV] disease resulting in infectious and parasitic diseases |  |  |
|  |  | Human immunodeficiency virus [HIV] disease resulting in malignant neoplasms |  |  |
|  |  | Human immunodeficiency virus [HIV] disease resulting in other conditions |  |  |
|  |  | Pulmonary tuberculosis |  |  |
|  |  | Respiratory tuberculosis, bacteriologically and histologically confirmed |  |  |
|  |  | Respiratory tuberculosis, not confirmed bacteriologically or histologically |  |  |
|  |  | Sequelae of tuberculosis |  |  |
|  |  | All other forms of tuberculosis |  |  |
|  |  | Unspecified TB AIDS |  |  |
|  |  | Tuberculosis of other organs |  |  |
|  |  | Miliary tuberculosis |  |  |
|  |  | Tuberculosis of nervous system |  |  |
|  |  | Unspecified human immunodeficiency virus [HIV] disease |  |  |
| *Non Communicable diseases* | Oral neoplasms | Malignant neoplasm of gum | Oral neoplasms |  |
|  |  | Malignant neoplasm of other and unspecified parts of tongue |  |  |
| 8 |  | Malignant neoplasm of other and unspecified parts of mouth |  |  |
| 9 | Digestive neoplasms | Carcinoma of gastrointestinal tract | Digestive neoplasms | Digestive neoplasms |
|  |  | Malignant neoplasm of colon |  |  |
|  |  | Malignant neoplasm of other and ill-defined digestive organs |  |  |
|  |  | Malignant neoplasm of small intestine |  |  |
|  |  | Malignant neoplasm of pancreas |  |  |
|  |  | Malignant neoplasm of stomach |  |  |
| 10 | Respiratory neoplasms | Carcinoma of the lung | Respiratory neoplasms | Respiratory neoplasms |
|  |  | Secondary malignant neoplasm of respiratory and digestive organs |  |  |
|  |  | Malignant neoplasm of other and ill-defined sites in the respiratory system and intrathoracic organs |  |  |
|  |  | Malignant neoplasm of bronchus and lung |  |  |
| 11 | Breast neoplasms | Carcinoma breast | Breast neoplasms | Breast neoplasms |
|  |  | Malignant neoplasm of breast |  |  |
| 12 | Reproductive neoplasms | Benign neoplasm of male genital organs | Reproductive neoplasms MF | Reproductive neoplasms MF |
|  |  | Carcinoma cervix or uterus |  |  |
|  |  | Malignant neoplasm of cervix uteri |  |  |
|  |  | Malignant neoplasm of corpus uteri |  |  |
|  |  | Malignant neoplasm of penis |  |  |
|  |  | Other benign neoplasms of uterus |  |  |
|  |  | Malignant neoplasm of other and unspecified female genital organs |  |  |
|  |  | Malignant neoplasm of prostate |  |  |
|  |  | Malignant neoplasm of testis |  |  |
| 13 | Other and unspecified neoplasms | Kaposi sarcoma | Other and unspecified neoplasms | Other and unspecified neoplasms |
|  |  | Other malignant neoplasms of skin |  |  |
|  |  | Malignant neoplasm of bladder |  |  |
|  |  | Malignant neoplasm of bone and articular cartilage of limbs |  |  |
|  |  | Malignant neoplasm of bone and articular cartilage of other and unspecified sites |  |  |
|  |  | Malignant neoplasm of liver and intrahepatic bile ducts |  |  |
|  |  | Malignant neoplasm of eye and adnexa |  |  |
|  |  | Malignant neoplasm of oesophagus |  |  |
|  |  | Secondary malignant neoplasm of respiratory and digestive organs |  |  |
|  |  | Malignant neoplasm of other and ill-defined sites |  |  |
|  |  | Secondary and unspecified malignant neoplasm of lymph nodes |  |  |
|  |  | Malignant neoplasm of other and unspecified parts of tongue |  |  |
|  |  | Malignant neoplasm of other connective and soft tissue |  |  |
|  |  | Malignant neoplasm, without specification of site |  |  |
|  |  | All other specified neoplasms |  |  |
|  |  | Leukaemia of unspecified cell type |  |  |
|  |  | Neoplasm of uncertain or unknown behaviour of other and unspecified sites |  |  |
|  |  | Unspecified neoplasms |  |  |
| 14 | Severe anaemia | Anaemia | Severe anaemia | Severe anaemia |
|  |  | Other nutritional anaemias |  |  |
|  |  | Other aplastic anaemias |  |  |
|  |  | Iron deficiency anaemia |  |  |
|  |  | Other anaemias |  |  |
|  |  | Acquired haemolytic anaemia |  |  |
|  |  | Acute posthaemorrhagic anaemia |  |  |
|  |  | Anaemia in chronic diseases classified elsewhere |  |  |
| 15 | Severe malnutrition | Malnutrition | Severe malnutrition | Severe malnutrition |
|  |  | Kwashiorkor |  |  |
|  |  | Marasmic kwashiorkor |  |  |
|  |  | Nutritional marasmus |  |  |
|  |  | Unspecified severe protein-energy malnutrition |  |  |
|  |  | Unspecified protein-energy malnutrition |  |  |
| 16 | Diabetes mellitus | Diabetes | Diabetes mellitus | Diabetes mellitus |
|  |  | Type 1 diabetes mellitus |  |  |
|  |  | Type 2 diabetes mellitus |  |  |
|  |  | Unspecified diabetes mellitus |  |  |
| 17 | Liver disease | Liver cirrhosis | Liver cirrhosis | Liver cirrhosis |
|  |  | Alcoholic liver disease |  |  |
|  |  | Alcoholic liver disease |  |  |
|  |  | All other specified liver diseases |  |  |
|  |  | Fibrosis and cirrhosis of liver |  |  |
|  |  | Other inflammatory liver diseases |  |  |
|  |  | Other diseases of liver |  |  |
|  |  | Hepatic failure, not elsewhere classified |  |  |
|  |  | Chronic viral hepatitis |  |  |
|  |  | Toxic liver disease |  |  |
|  |  | Oesophageal varices |  |  |
|  |  | Unspecified liver disease |  |  |
| 18 | Acute abdomen | Acute abdominal conditions | Acute abdomen | Acute abdomen |
|  |  | All other specified acute abdominal conditions |  |  |
|  |  | Acute pancreatitis |  |  |
|  |  | Duodenal ulcer |  |  |
|  |  | Peptic ulcer, site unspecified |  |  |
|  |  | Gastric ulcer |  |  |
|  |  | Gastritis and duodenitis |  |  |
|  |  | Other diseases of intestine |  |  |
|  |  | Gastro-oesophageal reflux disease |  |  |
|  |  | Other diseases of digestive system |  |  |
|  |  | Other gastroenteritis and colitis of infectious and unspecified origin |  |  |
|  |  | Other disorders of pancreatic internal secretion |  |  |
|  |  | CrohnÔÇÖs disease [regional enteritis] |  |  |
|  |  | Other noninfective gastroenteritis and colitis |  |  |
| 19 | Renal failure | Renal disorders | Renal failure | Renal failure |
|  |  | Acute renal failure |  |  |
|  |  | Chronic kidney disease |  |  |
|  |  | Unspecified kidney failure |  |  |
|  |  | Other disorders of kidney and ureter, not elsewhere classified |  |  |
|  |  | Hypertensive renal disease |  |  |
|  |  | Nephrotic syndrome |  |  |
|  |  | Specified renal disorders |  |  |
| 20 | Cardiac disease | Cardiac arrest | Acute cardiac disease | Acute cardiac disease |
|  |  | All other specified cardiovascular disorders | Other and unspecified cardiac disease | Other and unspecified cardiac disease |
|  |  | Heart failure | Stroke | Stroke |
|  |  | Hypertensive heart and renal disease | Chronic obstructive pulmonary disease | Chronic obstructive pulmonary disease |
|  |  | Hypertensive heart disease |  |  |
|  |  | Ischaemic heart disease |  |  |
|  |  | Chronic ischaemic heart disease |  |  |
|  |  | Essential (primary) hypertension |  |  |
|  |  | Congestive cardiac failure |  |  |
|  |  | Acute myocardial infarction |  |  |
|  |  | Acute pericarditis |  |  |
|  |  | Cerebral infarction |  |  |
|  |  | Cerebrovascular disease |  |  |
|  |  | Intracerebral haemorrhage |  |  |
|  |  | Myocarditis in diseases classified elsewhere |  |  |
|  |  | Occlusion and stenosis of precerebral arteries, not resulting in cerebral infarction |  |  |
|  |  | Other cerebrovascular diseases |  |  |
|  |  | Other diseases of pericardium |  |  |
|  |  | Other venous embolism and thrombosis |  |  |
|  |  | Sequelae of cerebrovascular disease |  |  |
|  |  | Subarachnoid haemorrhage |  |  |
|  |  | Cardiomyopathy |  |  |
|  |  | Pulmonary embolism |  |  |
|  |  | Essential (primary) hypertension |  |  |
|  |  | Complications and ill-defined descriptions of heart disease |  |  |
|  |  | Hypertension |  |  |
|  |  | Stroke, not specified as haemorrhage or infaction |  |  |
|  |  | Angina Pectoris |  |  |
|  |  | Secondary hypertension |  |  |
|  |  | Other acute ischaemic heart diseases |  |  |
|  |  | Chronic obstructive pulmonary disease |  |  |
|  |  | Other chronic obstructive pulmonary disease |  |  |
|  |  | Other pulmonary heart diseases |  |  |
|  |  | Unspecified cardiovascular disorders |  |  |
| 21 | Epilepsy | Epilepsy | Epilepsy | Epilepsy |
|  |  | Status epilepticus |  |  |
| 22 | Other and unspecified NCD | Sickle-cell disorders | Sickle cell with crisis | Sickle cell with crisis |
|  |  | Asthma | Asthma | Other and unspecified NCD |
|  |  | Abdominal and pelvic pain | Other and unspecified NCD |  |
|  |  | All other specified noncommunicable d.. |  |  |
|  |  | Complications and ill-defined descrip.. |  |  |
|  |  | Decubitus ulcer and pressure area |  |  |
|  |  | Headache |  |  |
|  |  | Other headache syndromes |  |  |
|  |  | Localized swelling, mass and lump of skin and subcutaneous tissue |  |  |
|  |  | Hepatoma |  |  |
|  |  | Cystic fibrosis |  |  |
|  |  | Hyperplasia of prostate |  |  |
|  |  | Mental and behavioural disorders due to use of alcohol |  |  |
|  |  | Other rheumatoid arthritis |  |  |
|  |  | Other symptoms and signs involving the circulatory and respiratory systems |  |  |
|  |  | Other symptoms and signs involving the digestive system and abdomen |  |  |
|  |  | Other disorders of veins |  |  |
|  |  | Other disorders of urinary system |  |  |
|  |  | Abnormalities of breathing |  |  |
|  |  | Adult respiratory distress syndrome |  |  |
|  |  | Bullous disorders in diseases classified elsewhere |  |  |
|  |  | Cerebral palsy |  |  |
|  |  | All other specified CNS disorders |  |  |
|  |  | Convulsions, not elsewhere classified |  |  |
|  |  | Cushing syndrome |  |  |
|  |  | Delirium, not induced by alcohol and other psychoactive substances |  |  |
|  |  | Decubitus ulcer and pressure area |  |  |
|  |  | Depressive episode |  |  |
|  |  | Effects of other deprivation |  |  |
|  |  | Diseases of pulp and periapical tissues |  |  |
|  |  | Disseminated intravascular coagulation [defibrination syndrome] |  |  |
|  |  | Haemorrhage from respiratory passages |  |  |
|  |  | Erythema multiforme |  |  |
|  |  | Inflammatory polyneuropathy |  |  |
|  |  | Intracranial and intraspinal abscess and granuloma |  |  |
|  |  | Migraine |  |  |
|  |  | Multisystem inflammatory syndrome associated with COVID-19, unspecified |  |  |
|  |  | Myositis |  |  |
|  |  | Other diseases of biliary tract |  |  |
|  |  | Other disorders involving the immune mechanism, not elsewhere classified |  |  |
|  |  | Other dermatitis |  |  |
|  |  | Other disorders of brain |  |  |
|  |  | Other disorders of fluid, electrolyte and acid-base balance |  |  |
|  |  | Other disorders of central nervous system |  |  |
|  |  | Other disorders of nervous system in .. |  |  |
|  |  | Other polyneuropathies |  |  |
|  |  | Other sudden death, cause unknown |  |  |
|  |  | Pleural effusion, not elsewhere classified |  |  |
|  |  | Postprocedural disorders of circulatory system, not elsewhere classified |  |  |
|  |  | Postprocedural disorders of genitourinary system, not elsewhere classified |  |  |
|  |  | Schizophrenia |  |  |
|  |  | Senility |  |  |
|  |  | Shock, not elsewhere classified |  |  |
|  |  | Status asthmaticus |  |  |
|  |  | Stomatitis and related lesions |  |  |
|  |  | Spina bifida |  |  |
|  |  | Symptoms and signs concerning food and fluid intake |  |  |
|  |  | Syncope and collapse |  |  |
|  |  | Toxic encephalopathy |  |  |
|  |  | Volume depletion |  |  |
|  |  | Paralytic ileus and intestinal obstru.. |  |  |
|  |  | Postprocedural disorders of circulato.. |  |  |
|  |  | Central nervous system disorders |  |  |
|  |  | Fracture of spine, level unspecified |  |  |
|  |  | Other injuries of spine and trunk, level unspecified |  |  |
|  |  | Unspecified dementia |  |  |
|  |  | Inguinal hernia |  |  |
|  |  | Umbilical hernia |  |  |
|  |  | Unspecified abdominal hernia |  |  |
|  |  | Unspecified noncommunicable causes |  |  |
|  |  | Unspecified organic or symptomatic mental disorder |  |  |
|  |  | Other respiratory disorders |  |  |
|  |  | Respiratory failure, not elsewhere classified |  |  |
| *Maternal causes* | Abortion-related death | Abortion |  | Abortion-related death |
| 23 |  | Specified abortion |  |  |
|  |  | Spontaneous abortion |  |  |
|  |  | Other abortion |  |  |
|  |  | Unspecified abortion |  |  |
|  |  | Failed attempted abortion |  |  |
| 24 | Obstetric haemorrhage | Postpartum haemorrhage | Obstetric haemorrhage | Obstetric haemorrhage |
|  |  | Specified ante or postpartum haemorrhage |  |  |
|  |  | Ante or postpartum haemorrhage |  |  |
| 25 | Other and unspecified maternal CoD | Eclampsia | Pregnancy-induced hypertension | Pregnancy-induced hypertension |
|  |  | Pre-eclampsia | Obstructed labour | Pregnancy-related sepsis |
|  |  | Obstructive and reflux uropathy | Pregnancy-related sepsis | Anaemia of pregnancy |
|  |  | Other obstructed labour | Ruptured uterus | Ruptured uterus |
|  |  | Other specified direct maternal causes | Other and unspecified maternal CoD |  |
|  |  | Ectopic pregnancy | Congenital malformation |  |
|  |  | Puerperal sepsis |  |  |
|  |  | Birth injury and or asphyxia |  |  |
|  |  | Bacterial sepsis of newborn |  |  |
|  |  | Unspecified direct maternal causes |  |  |
|  |  | Complications of anaesthesia during pregnancy |  |  |
|  |  | Congenital abnormalities |  |  |
|  |  | Obstructed labour |  |  |
|  |  | Prematurity and or low birth weight |  |  |
|  |  | Other obstetric trauma |  |  |
|  |  | Obstetric death of unspecified cause |  |  |
|  |  | Maternal infectious and parasitic diseases classifiable elsewhere but complicating pregnancy, childbirth and the puerperium |  |  |
|  |  | Other maternal diseases classifiable elsewhere but complicating pregnancy, childbirth and the puerperium |  |  |
|  |  | Complications of anaesthesia during labour and delivery |  |  |
|  |  | Other abnormal uterine and vaginal bleeding |  |  |
|  |  | Still birth |  |  |
|  |  | Other puerperal infections |  |  |
|  |  | Other congenital malformations of heart |  |  |
|  |  | All other perinatal causes |  |  |
| *External Causes* | Transport Accident | Bus occupant injured in other and unspecified transport accidents | Road traffic accident | Road traffic accident |
| 26 |  | Car occupant injured in collision with fixed or stationary object | Other transport accident | Other transport accident |
|  |  | Car occupant injured in collision with heavy transport vehicle or bus |  |  |
|  |  | Motor- or nonmotor-vehicle accident, type of vehicle unspecified |  |  |
|  |  | Motorcycle rider injured in collision with car, pick-up truck or van |  |  |
|  |  | Motorcycle rider injured in collision with heavy transport vehicle or bus |  |  |
|  |  | Motorcycle rider injured in collision with two- or three-wheeled motor vehicle |  |  |
|  |  | Motorcycle rider injured in noncollision transport accident |  |  |
|  |  | Motorcycle rider injured in other and unspecified transport accidents |  |  |
|  |  | Occupant of heavy transport vehicle injured in collision with pedal cycle |  |  |
|  |  | Occupant of heavy transport vehicle injured in noncollision transport accident |  |  |
|  |  | Occupant of heavy transport vehicle injured in collision with heavy transport vehicle or bus |  |  |
|  |  | Occupant of pick-up truck or van injured in collision with fixed or stationary object |  |  |
|  |  | Pedal cyclist injured in collision with heavy transport vehicle or bus |  |  |
|  |  | Pedal cyclist injured in non collision transport accident |  |  |
|  |  | Pedestrian injured in collision with two- or three-wheeled motor vehicle |  |  |
|  |  | Pedestrian injured in other and unspecified transport accidents |  |  |
|  |  | Unspecified transport accident |  |  |
|  |  | Traffic accident of specified type but victimÔÇÖs mode of transport unknown |  |  |
|  |  | Road traffic accident |  |  |
| 27 | Accid poisoning & noxious subs | Accidental poisoning |  |  |
|  |  | Toxic effect of pesticides |  |  |
|  |  | Toxic effect of other and unspecified substances |  |  |
|  |  | Poisoning by and exposure to alcohol, undetermined intent |  |  |
|  |  | Respiratory conditions due to inhalation of chemicals, gases, fumes and vapours |  |  |
|  |  | Respiratory conditions due to other external agents |  |  |
|  |  | Toxic effect of alcohol |  |  |
|  |  | Accidental poisoning by and exposure to pesticides |  |  |
|  |  | Poisoning by and exposure to other and unspecified chemicals and noxious substances, undetermined intent |  |  |
| 28 | Contact with venomous plant/animal | Animal Bite or Attack | Contact with venomous plant/animal | Contact with venomous plant/animal |
|  |  | Bitten or struck by other mammals |  |  |
|  |  | Toxic effect of contact with venomous animals |  |  |
|  |  | Rat-bite fevers |  |  |
|  |  | Animal-rider or occupant of animal-drawn vehicle injured in transport accident |  |  |
| 29 | Assault | Assault by blunt object | Assault | Assault |
|  |  | Assault by hanging, strangulation and suffocation |  |  |
|  |  | Assault by sharp object |  |  |
|  |  | Assault by unspecified means |  |  |
| 30 | Accid drowning and submersion | Drowning | Accid drowning and submersion | Accid drowning and submersion |
|  |  | Accident to watercraft causing drowning and submersion |  |  |
|  |  | Unspecified drowning and submersion |  |  |
|  |  | Other specified drowning and submersion |  |  |
|  |  | Diving or jumping into water causing injury other than drowning or submersion |  |  |
|  |  | Drowning and submersion while in natural water |  |  |
| 31 | Accid expos to smoke fire & flame | Burn and corrosion of trunk | Accid expos to smoke fire & flame | Accid expos to smoke fire & flame |
|  |  | Burn and corrosion, body region unspecified |  |  |
|  |  | Burns |  |  |
|  |  | Exposure to uncontrolled fire in building or structure |  |  |
| 32 | Intentional self-harm | Intentional injuries | Intentional self-harm | Intentional self-harm |
|  |  | Suicidal injuries |  |  |
|  |  | Homicidal injuries |  |  |
|  |  | Intentional self-harm by hanging, strangulation and suffocation |  |  |
|  |  | Intentional self-harm by unspecified means |  |  |
|  |  | Intentional self-harm by drowning and submersion |  |  |
|  |  | Intentional self-poisoning by and exposure to alcohol |  |  |
|  |  | Intentional self-poisoning by and exposure to other and unspecified chemicals and noxious substances |  |  |
|  |  | Intentional self-poisoning by and exposure to pesticides |  |  |
| 33 | Accid fall | Fall from tree | Accid fall | Accid fall |
|  |  | Fall involving bed |  |  |
|  |  | Unspecified fall |  |  |
|  |  | Fall on same level from slipping, tripping and stumbling |  |  |
|  |  | Falls |  |  |
| 34 | Other and unspecified external CoD | Crushing injuries involving multiple body regions | Other and unspecified external CoD | Other and unspecified external CoD |
|  |  | Exposure to other and unspecified forces of nature | Exposure to other and unspecified forces of nature | Exposure to other and unspecified forces of nature |
|  |  | Crushing injury of head |  |  |
|  |  | Exposure to unspecified electric current |  |  |
|  |  | Effects of other external causes |  |  |
|  |  | Fractures involving multiple body regions |  |  |
|  |  | Injury of other and unspecified intrathoracic organs |  |  |
|  |  | Hanging, strangulation and suffocation, undetermined intent |  |  |
|  |  | Injury of unspecified body region |  |  |
|  |  | Other and unspecified injuries of abdomen, lower back and pelvis |  |  |
|  |  | Other maltreatment |  |  |
|  |  | Maltreatment syndromes |  |  |
|  |  | Asphyxiation |  |  |
|  |  | Complications of procedures, not elsewhere classified |  |  |
|  |  | Certain early complications of trauma, not elsewhere classified |  |  |
|  |  | Injury of unspecified body region |  |  |
|  |  | Failure of sterile precautions during surgical and medical care |  |  |
|  |  | Intracranial injury |  |  |
|  |  | Other and unspecified injuries of head |  |  |
|  |  | Other and unspecified injuries of thorax |  |  |
|  |  | Other complications of surgical and medical care, not elsewhere classified |  |  |
|  |  | Unspecified threat to breathing |  |  |
|  |  | Traumatic amputation at neck level |  |  |
|  |  | Other specified unintentional injuries |  |  |
|  |  | Sequelae of injuries involving multiple and unspecified body regions |  |  |
|  |  | Sequelae of injuries of head |  |  |
|  |  | Sequelae of injuries of lower limb |  |  |
|  |  | Sequelae of injuries of neck and trunk |  |  |
|  |  | Sequelae of other and unspecified effects of external causes |  |  |
|  |  | Unspecified external causes |  |  |
|  |  | Unspecified unintentional injuries |  |  |
|  |  | Unspecified multiple injuries |  |  |
| *Other and undetermined Causes* | Other causes of death not classified | Adverse effects, not elsewhere classi.. |  |  |
| 35 |  | All other specified symptoms, signs a.. |  |  |
|  |  | Emergency use of U07 |  |  |
|  |  | Other ill-defined and unspecified causes of mortality |  |  |
|  |  | Pain in throat and chest |  |  |
| 36 | Undetermined | Undetermined |  |  |
| 37 | Unattended death | Unattended death |  |  |
