# Supplementary material for: A comparison of verbal autopsy assignment methods to obtain adult cause-specific mortality in two longitudinal studies in Rakai and Kalungu districts of South Central, Uganda
Source: PLOS Glob Public Health. 2026 Apr 6;6(4):e0006223. doi: 10.1371/journal.pgph.0006223 (PMC13052855; doi:10.1371/journal.pgph.0006223)
Supplement: S2 Table — (DOCX) [file pgph.0006223.s002.docx]

**S2 Table: Classification of undetermined Physician CoD (N=236) by the algorithms**

| **Cause of death** | **InterVA-5** | **InSilicoVA** |
| --- | --- | --- |
| Infectious and parasitic diseases | 78(33.05) | 103(43.64) |
| Neoplasms | 37(15.68) | 19(8.05) |
| Nutrtional and endocrine disorders | 8(3.39) | 3(1.27) |
| Diseases of the circulatory system | 47(19.92) | 39(16.53) |
| Respiratory disorders | 1(0.42) | 0(0) |
| Gastrointestinal disorders | 6(2.54) | 9(3.81) |
| Renal disorders | 7(2.97) | 12(5.08) |
| Epilepsy | 12(5.08) | 8(3.39) |
| Pregnancy, childbirth and puerperium-re | 9(3.81) | 6(2.54) |
| External causes of death | 24(10.17) | 28(11.86) |
| Cause of death unknown | 7(2.97) | 9(3.81) |
