# Supplementary material for: A comparison of verbal autopsy assignment methods to obtain adult cause-specific mortality in two longitudinal studies in Rakai and Kalungu districts of South Central, Uganda
Source: PLOS Glob Public Health. 2026 Apr 6;6(4):e0006223. doi: 10.1371/journal.pgph.0006223 (PMC13052855; doi:10.1371/journal.pgph.0006223)
Supplement: S3 Table — (DOCX) [file pgph.0006223.s003.docx]

**S3 Table:** **A mapping of the ICD-10 codes to the WHO CoD list and Broad CoD list as used in this study.**

| **ICD-10 codes (from ICD)** | **Verbal autopsy title** | **WHO VA category** | **Broad Cause** |
| --- | --- | --- | --- |
| A40-A41 | Sepsis | **Infectious and parasitic diseases** | **Other Communicable causes** |
| J00-J22 | Acute respiratory infection, including pneumonia |  |  |
| A00-A09 | Diarrheal diseases |  |  |
| B50-B54 | Malaria |  |  |
| B05 | Measles |  |  |
| A39; G00- G05 | Meningitis and encephalitis |  |  |
| A33-A35 | Tetanus (Excludes: Neonatal tetanus A34) |  |  |
| A37 | Pertussis |  |  |
| A90-A99 | Haemorrhagic fever |  |  |
| A17-A19 A20-A38; A42-A89; B00-B19; B25-B49; B55-B99 | Other and unspecified infectious disease |  |  |
|  |  |  |  |
| B20-B24 | HIV/AIDS related death | **HIV/TB** | **HIV/TB** |
| A15-A16 | Pulmonary tuberculosis |  |  |
|  |  |  |  |
| D55-D89; E00-E07; E15-E35; E50-E90; F00-F99; G06-G09 G10-G37; G50-G99; H00-H95; J30-J39; J47-J99; K00-K31; K35-K38 K40-K93; L00-L99; M00-M99; N00-N16; N20-N99; R00-R09 R11-R94 | Other and unspecified non- communicable disease | **Other and unspecified non Communicable Causes** | **Non-Communicable causes** |
|  |  |  |  |
| C00-C06 | Oral neoplasms | **Neoplasms** |  |
| C15-C26 | Digestive neoplasms |  |  |
| C30-C39 | Respiratory neoplasms |  |  |
| C50 | Breast neoplasms |  |  |
| C51-C58 | Female reproductive neoplasms |  |  |
| C60-C63 | Male reproductive neoplasms |  |  |
| C07-C14 C40-C49 | Other and unspecified neoplasms |  |  |
|  |  |  |  |
| D50-D64 | Severe anaemia | **Nutritional and endocrine disorders** |  |
| E40-E46 | Severe malnutrition |  |  |
| E10-E14 | Diabetes mellitus |  |  |
|  |  |  |  |
| I20-I25 | Acute cardiac disease | **Diseases of the circulatory system** |  |
| I60-I69 | Stroke |  |  |
| D57 | Sickle cell with crisis |  |  |
| I00-I09 I10-I15 I26-I52 I70-I99 | Other and unspecified cardiac disease |  |  |
|  |  |  |  |
| J40-J44 | Chronic obstructive pulmonary disease (COPD) | **Respiratory disorders** |  |
| J45-J46 | Asthma |  |  |
|  |  |  |  |
| R10 | Acute abdomen | **Gastrointestinal disorders** |  |
| K70-K76 | Liver cirrhosis |  |  |
|  |  |  |  |
| N17-N19 | Renal failure | **Renal disorders** |  |
|  |  |  |  |
| G40-G41 | Epilepsy | **Epilepsy** |  |
|  |  |  |  |
| O00 | Ectopic pregnancy | **Pregnancy, childbirth and puerperium -related disorders** | **Maternal Causes** |
| O03-O08 | Abortion-related death |  |  |
| O10-O16 | Pregnancy-induced hypertension |  |  |
| O46; O67; O72 | Obstetric haemorrhage |  |  |
| O63-O66 | Obstructed labour |  |  |
| O85; O75.3 | Pregnancy-related sepsis |  |  |
| O99.0 | Anaemia of pregnancy |  |  |
| O71 | Ruptured uterus |  |  |
| O01-O02; O20-O45; O47-O62; O68-O70; O73-O84; O86-O99 | Other and unspecified maternal cause |  |  |
|  |  |  |  |
| V01-V89 | Road traffic accident | **External Causes of death** | **External Causes** |
| V90-V99 | Other transport accident |  |  |
| W00-W19 | Accidental fall |  |  |
| W65-W74 | Accidental drowning and submersion |  |  |
| X00-X19 | Accidental exposure to smoke, fire and flames |  |  |
| X20-X29 | Contact with venomous animals and plants |  |  |
| X40-X49 | Accidental poisoning and exposure to a noxious substance |  |  |
| X60-X84 | Intentional self-harm |  |  |
| X85-Y09 | Assault |  |  |
| X30-X39 | Exposure to the force of nature |  |  |
| S00-T99; W20-W64; W75-W99; X50-X59; Y10-Y98 | Other and unspecified external cause of death |  |  |
|  |  |  |  |
| R95-R99 | Cause of death unknown | **Unknown causes** | **Undetermined causes** |
