# Supplementary material for: A comparison of verbal autopsy assignment methods to obtain adult cause-specific mortality in two longitudinal studies in Rakai and Kalungu districts of South Central, Uganda
Source: PLOS Glob Public Health. 2026 Apr 6;6(4):e0006223. doi: 10.1371/journal.pgph.0006223 (PMC13052855; doi:10.1371/journal.pgph.0006223)
Supplement: S4 Table — (DOCX) [file pgph.0006223.s004.docx]

**S4 Table. Comparison of InterVA-5 and InSilicoVA with Physician Reviews in Rakai, without Application of the 0.4 Cutoff on probability of CoD assigned by the algorithms**

|  | **Individual-level agreement** | | |  | **Population-level agreement** | |
| --- | --- | --- | --- | --- | --- | --- |
|  | **Overall percentage agreement (95% CI)** | **Overall percentage agreement - WHO cause list (95% CI)** | **Overall percentage agreement (Top cause - WHO cause list) (95% CI)** |  | **CSMF - Accuracy (95% CI)** | **Spearman's correlation (95% CI)** |
| **Total Sample N=702** |  |  |  |  |  |  |
| InterVA-5 | 40.59(37.02-44.28) | 41.31(37.72-45.00) | 12.54(10.28-15.20) |  | 0.89(0.86-0.91) | 0.52(0.46-0.57) |
| InSilico VA | 38.89(35.34-42.56) | 45.16(41.50-48.86) | 11.82(9.63-14.43) |  | 0.83(0.79-0.87) | 0.55(0.49-0.59) |
| **Males n=377** |  |  |  |  |  |  |
| InterVA-5 | 39.52(34.69-44.57) | 44.03(39.08-49.10) | 12.99(9.95-16.79) |  | 0.88(0.85–0.92) | 0.57(0.49 - 0.63) |
| InSilico VA | 36.60(31.88-41.61) | 46.42(41.42-51.49) | 12.20(9.26-15.92) |  | 0.81(0.77–0.86) | 0.56(0.49- 0.63) |
| **Females n=325** |  |  |  |  |  |  |
| InterVA-5 | 41.85(36.58-47.31) | 38.15(33.01-43.58) | 12.00(8.88-16.02) |  | 0.88(0.84–0.92) | 0.46(0.36-0.54) |
| InSilico VA | 41.54(36.28-46.99) | 43.69(38.37-49.16) | 11.69(8.62-15.68) |  | 0.86(0.79–0.91) | 0.52(0.44-0.59) |
| **15-49 years n=276** |  |  |  |  |  |  |
| InterVA-5 | 52.19(46.25-58.07) | 57.29(51.34-63.05) | 25.18(20.38-30.68) |  | 0.86(0.82-0.91) | 0.70(0.63 - 0.75) |
| InSilico VA | 48.54(42.64-54.48) | 60.58(54.65-66.22) | 22.99(18.37-28.37) |  | 0.84(0.79–0.89) | 0.75(0.69 - 0.79) |
| **50+ years n=426** |  |  |  |  |  |  |
| InterVA-5 | 33.18(28.87-37.79) | 31.07(26.86-35.63) | 6.07(4.16-8.78) |  | 0.89(0.86–0.93) | 0.36(0.28 - 0.44) |
| InSilico VA | 32.71(28.42-37.31) | 35.28(30.88-39.94) | 12.38(9.58-15.87) |  | 0.83(0.78–0.88) | 0.38(0.29 - 0.46) |
